# Applications of Nanomaterials Based on Magnetite and Mesoporous Silica on the Selective Detection of Zinc Ion in Live Cell Imaging

**DOI:** 10.3390/nano8060434

**Published:** 2018-06-14

**Authors:** Roghayeh Sadeghi Erami, Karina Ovejero, Soraia Meghdadi, Marco Filice, Mehdi Amirnasr, Antonio Rodríguez-Diéguez, María Ulagares De La Orden, Santiago Gómez-Ruiz

**Affiliations:** 1 Department of Chemistry, Isfahan University of Technology, Isfahan 84156-83111, Iran; romochem@gmail.com (R.S.E.); smeghdad@cc.iut.ac.ir (S.M.); 2 Departamento de Biología y Geología, Física y Química Inorgánica, ESCET, Universidad Rey Juan Carlos, Calle Tulipán s/n, E-28933 Móstoles, Madrid, Spain; 3 National Research Centre for Cardiovascular Disease (CNIC), Melchor Fernández Almagro, 3, 28029 Madrid, Spain; k.ovejero94@gmail.com; 4 Department of Chemistry in Pharmaceutical Sciences, Faculty of Pharmacy, Complutense University (UCM), Plaza Ramón y Cajal s/n, 28040 Madrid, Spain; 5 Biomedical Research Networking Center for Respiratory Diseases (CIBERES), Melchor Fernández Almagro, 3, 28029 Madrid, Spain; 6 Departamento de Química Inorgánica, Facultad de Ciencias, Campus de Fuentenueva. Avda. Fuentenueva s/n, 18071 Granada, Spain; antonio5@ugr.es; 7 Departamento de Química Orgánica I, E. U. Óptica, Universidad Complutense de Madrid, Arcos de Jalón, s/n, 28037 Madrid, Spain; mariula@quim.ucm.es

**Keywords:** nanomaterials, mesoporous silica, magnetite, live cell imaging, Zn^2+^ detection, Zn^2+^ sensors

## Abstract

Functionalized magnetite nanoparticles (FMNPs) and functionalized mesoporous silica nanoparticles (FMSNs) were synthesized by the conjugation of magnetite and mesoporous silica with the small and fluorogenic benzothiazole ligand, that is, 2(2-hydroxyphenyl)benzothiazole (**hpbtz**). The synthesized fluorescent nanoparticles were characterized by FTIR, XRD, XRF, ^13^C CP MAS NMR, BET, and TEM. The photophysical behavior of FMNPs and FMSNs in ethanol was studied using fluorescence spectroscopy. The modification of magnetite and silica scaffolds with the highly fluorescent benzothiazole ligand enabled the nanoparticles to be used as selective and sensitive optical probes for zinc ion detection. Moreover, the presence of **hpbtz** in FMNPs and FMSNs induced efficient cell viability and zinc ion uptake, with desirable signaling in the normal human kidney epithelial (Hek293) cell line. The significant viability of FMNPs and FMSNs (80% and 92%, respectively) indicates a potential applicability of these nanoparticles as in vitro imaging agents. The calculated limit of detections (LODs) were found to be 2.53 × 10^−6^ and 2.55 × 10^−6^ M for Fe_3_O_4_-H@hpbtz and MSN-Et_3_N-IPTMS-hpbtz-f1, respectively. FMSNs showed more pronounced zinc signaling relative to FMNPs, as a result of the more efficient penetration into the cells.

## 1. Introduction

The synthesis of fluorogenic and chromogenic probes with structural simplicity, high selectivity, and sensitivity for essential metal ions has attracted much interest because of the significance of cations in a physiological medium as well as human health. Among different metal ions, zinc is a vital structural component and is involved in the biological activity of many proteins, like enzymes receptors, and transcription factors corresponding to cellular signaling pathways [1,2,3,4].

Considering the function of zinc(II) ions as a signaling species in the intra- and inter-cellular processes, it is desirable to develop simple methods for the measurement of total bound and unbound zinc concentration in biological systems. Among various analytical methods for zinc ion measurement, much effort has recently been devoted to fluorescence microscopy or spectroscopy utilizing low molecular weight chelating agents or probes [5,6]. The contribution of probes and nanotechnology have been extensively considered in high-resolution cell imaging in single cells for defining the biochemical performances of zinc ions in cellular regulation [7,8,9,10,11,12,13].

With this concept in mind, the development of a method for the preparation of appropriate functionalized magnetite nanoparticles (FMNPs) with biological applications is an important issue. Generally, the surfaces activation of magnetic nanoparticles is provided by a post surface modification (PSM). The covalent binding of biomolecules, such as proteins and peptides, to the surface of magnetic nanoparticles by PSM has been frequently and extensively reported [14,15,16].

In addition to FMNPs, functionalized mesoporous silica nanoparticles (FMSNs) with specific properties, such as high specific surface area, large and regular pore size, high hydrothermal and photo stability, as well as biocompatibility, have also been considered in various fields, particularly in the fabrication of organic-inorganic hybrid imaging probes [17,18,19]. Usually, formulation of a mesoporous silica with covalently linked functionality is possible with two different strategies, post-synthesis modification, and direct or co-condensation synthesis [20].

In this context, our group was interested in the preparation of modified nanoparticles, including fluorescent benzothiazole ligands that were covalently bound to the material. We have chosen fluorescent benzothiazole derivatives as they are good candidates for the functionalization of nanoparticles because of their biocompatibility. For example, the photophysical, photochemical, and biological properties of 2-(2-hydroxyphenyl)benzothiazole (**hpbtz**) have been studied as antitumor, anti-inflammatory, and as positron-emission tomography (PET) imaging in the diagnosis of Alzheimer’s disease [21,22,23], and there are a good number of reports on reactive probes based on the **hpbtz** moiety for the selective detection of different analytes, such as Zn^2+^ ion [24,25,26,27,28,29,30,31,32,33]. However, several probes are prepared under harsh conditions or by using multi-step procedures [31,32,34].

A combination of the nanostructured materials with **hpbtz** derivatives of a small molecular structure and low molecular weight, along with facile preparation and high sensitivity, may provide functional nanomedical scaffolds for multimodal imaging or simultaneous diagnosis and therapy. Theoretical studies on the incorporation of **hpbtz** analogous as an interesting fluorescent guest molecule in nanosized zeolites and silica-based hybrid materials has already been reported [18,35,36]. However, the direct covalent functionalization of MNPs with benzothiazole ligands and application as Zn detecting agent in cell imaging, remains to be explored.

Inspired by the aforementioned reports and following our ongoing research on the synthesis of small, simple fluorogenic molecules and their application as zinc live-cell markers [37,38], herein, we report the synthesis and characterization of functionalized magnetite and mesoporous silica fluorescent nanoparticles (FMNPs and FMSNs), using 2-(2-hydroxyphenyl)benzothiazole (**hpbtz**) as the fluorogenic agent. We used two different nanoparticles (silica and magnetite) because magnetite-based materials, because of their magnetic properties, led to oversized aggregates of FMNPs and their internalization into the living cell was not adequate.

Thus, as an alternative to the MNPs, the incorporation of **hpbtz** into mesoporous silica nanoparticles (MSNs), having a favorable large surface area and pore volume for immobilization of the fluorophore species, was examined. Our biological results show the analysis of the in vitro zinc uptake of the synthesized materials in the presence of living HEK293 cells, which was confirmed by fluorescence confocal microscopy (CLSM). Our results indicate that the control of the particle size and the increase of the ligand surface functionalization play a critical role in the internalization and subcellular localization of the synthesized materials to improve and modulate the cytotoxic properties and zinc uptake in cellular response.

## 2. Materials and Methods

### 2.1. Materials

Iron(III) chloride hexahydrate 98%, iron(II) chloride tetrahydrate 99%, tetraethylortosilicate (TEOS), 98%,hexadecyltrimethylammonium bromide (CTAB), 2-hydroxybenzoic acid, triphenyl phosphite (TPP), tetrabutylammonium bromide (TBAB), 2-aminothiophenol, sodium hydroxide (NaOH), pyridine, 3-isocyanatopropyltriethoxysilane (ICTES), 3-iodopropyltrimethoxysilane (IPTMS), magnesium sulfate, and the nitrate salts of metal cations for fluorescence measurements, that is, Na^+^, K^+^, Ag^+^, Ca^2+^, Mg^2+^, Fe^2+^, Co^2+^, Ni^2+^, Cu^2+^, Zn^2+^, Cd^2+^, Ba^2+^, Hg^2+^, Pb^2+^, and Al^3+^, were purchased from Sigma Aldrich (Tres Cantos, Spain) and were used as received. Iron(III) nitrate nonahydrate, extra pure sodium acetate anhydrous, sodium hydrogen carbonate, ascorbic acid, and ethylene glycol were purchased from Scharlau (Móstoles, Spain) and were used as received, without further purification. Triethylamine (Et_3_N) redistilled 99% was purchased from Fluka (Tres Cantos, Spain) and was used as supplied. Organic solvents such as THF and acetone were purchased from Carlo Erba (Sabadell, Spain) and were distilled and dried over appropriate drying agents. Ethanol was purchased from VWR (Barcelona, Spain) and Milli-Q (Merck-Millipore, Barlington, MA, USA) quality water was used in the experiments.

### 2.2. Preparation of Functionalized Mesoporous Silica Nanoparticles (FMSNs)

#### 2.2.1. Synthesis of Mesoporous Silica Nanoparticles (MSNs)

The mesoporous silica nanoparticles (MSNs) were prepared following a previously reported method [39]. In a typical synthesis, hexadecyltrimethylammonium bromide (CTAB, 2.00 g, 5.48 mmol) and 7 mL of 2.00 M sodium hydroxide aqueous solution were added to a quantity of 960 mL of nanopure water. The mixture was then stirred and the temperature increased to 80 °C. Afterwards, the silicon source, tetraethylortosilicate (TEOS, 10 mL, 45 mmol), was slowly added dropwise to the previously heated solution, under vigorous stirring. The reaction mixture was then stirred for 2 additional hours after the addition of TEOS and the mixture gave a white precipitate. The mesoporous silica nanoparticles were then isolated by filtration, washed with nanopure water and methanol, and dried under vacuum overnight. The last step of was the surfactant template (CTAB), which was carried out by a simple calcination in air at 550 °C for 12 h.

#### 2.2.2. Synthesis of e1–e3 in the Presence of Different Bases

**Scheme 1:**
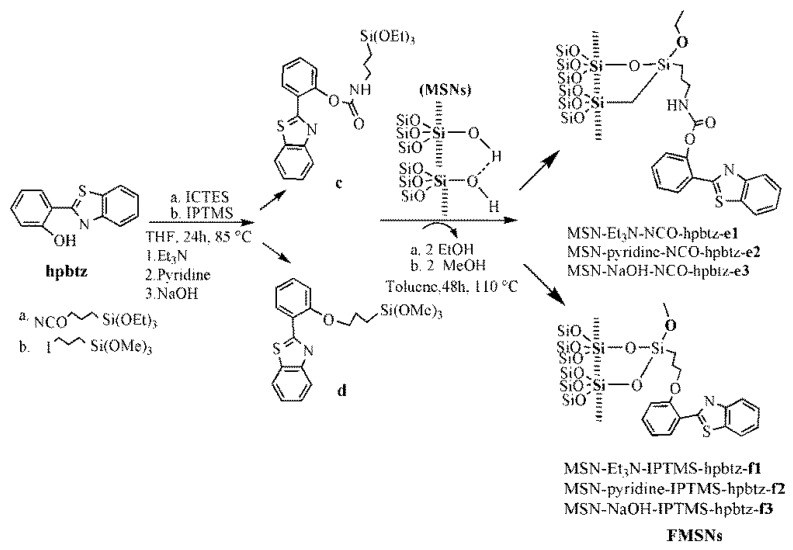
Synthesis of functionalized mesoporous silica nanoparticles (FMSNs). Reaction of benzothiazole ligand (**hpbtz**) with 3-isocyanatopropyltriethoxysilane (ICTES) and 3-iodopropyltrimethoxysilane (IPTMS) generates **c** and **d** in the presence of different bases. The reaction of the post functionalized materials with ICTES and IPTMS under a nitrogen atmosphere with MSNs, forms the **hpbtz** loaded materials, **e1–e3** and **f1–f3**, respectively. The resulting products are labeled as MSN-Et_3_N-IPTMS-hpbtz-**f1**, MSN-pyridine-IPTMS-hpbtz-**f2**, MSN-NaOH-IPTMS-hpbtz-**f3**, MSN-Et_3_N-NCO-hpbtz-**e1**, MSN-pyridine-NCO-hpbtz-**e2**, and MSN-NaOH-NCO-hpbtz-**e3**.

To functionalize the MSN with **hpbtz** (0.5 g, 2.2 mmol) in THF (15 mL), a solution of triethylamine (1 mL, 5 mmol) and then 3-isocyanatopropyltriethoxysilane (ICTES) (0.5 mL, 2 mmol) were added under a nitrogen atmosphere. The yellowish reaction mixture was stirred at 85 °C for 24 h to give **c**. After removing the solvent under the vacuum, 0.5 g of previously dehydrated (at 110 °C in vacuum for 12 h) MSNs were added to the above mixture in 15 mL toluene. The suspension was stirred at 110 °C for 48 h and the resulting light-yellow solid was then separated by filtration and washed with THF (4 × 10 mL) and acetone (1 × 15 mL). The same procedure was applied to the reaction of **hpbtz** with ICTES in the presence of pyridine (0.2 mL, 2.5 mmol) and sodium hydroxide (0.05 g, 1.25 mmol). The light yellowish solid was dried under a vacuum at 60 °C and stored under an inert atmosphere. The materials that were obtained in the presence of triethylamine, pyridine, and sodium hydroxide were labeled as MSN-Et_3_N-NCO-hpbtz-e1, MSN-pyridine-NCO-hpbtz-e2, and MSN-NaOH-NCO-hpbtz-e3, respectively (Scheme 1).

#### 2.2.3. Synthesis of f1–f3 in the Presence of Different Bases

To functionalize the nanomaterials with **hpbtz** (0.5 g, 2.2 mmol) in THF (15 mL), a solution of triethylamine (1 mL, 5 mmol) and, subsequently, 3-iodopropyltrimethoxysilane (IPTMS) (0.34 mL, 2 mmol) were added under a nitrogen atmosphere. The yellowish reaction mixture was stirred at 85 °C for 24 h for the preparation of **d**. After removing the solvent, 0.5 g of previously dehydrated (at 110 °C in vacuum for 12 h) MSNs were added to the above mixture, in 15 mL toluene. The suspension was stirred at 110 °C for 48 h and the resulting light-yellow solid was then separated by filtration and washed with tetrahydrofuran (4 × 10 mL) and acetone (1 × 15 mL). The same procedure was applied to the reaction of **hpbtz** with IPTMS in the presence of pyridine (0.2 mL, 2.5 mmol) and sodium hydroxide (0.05 g, 1.25 mmol). The light-yellowish solid was dried under a vacuum at 60 °C and was stored under an inert atmosphere. The compounds that were obtained in the presence of triethylamine, pyridine, and sodium hydroxide were labeled as MSN-Et_3_N-IPTMS-hpbtz-**f1**, MSN-pyridine-IPTMS-hpbtz-**f2**, and MSN-NaOH-IPTMS-hpbtz-**f3**, respectively (Scheme 1).

### 2.3. Detailed Synthesis Methodology of MNPs and FMNPs

**Scheme 2:**
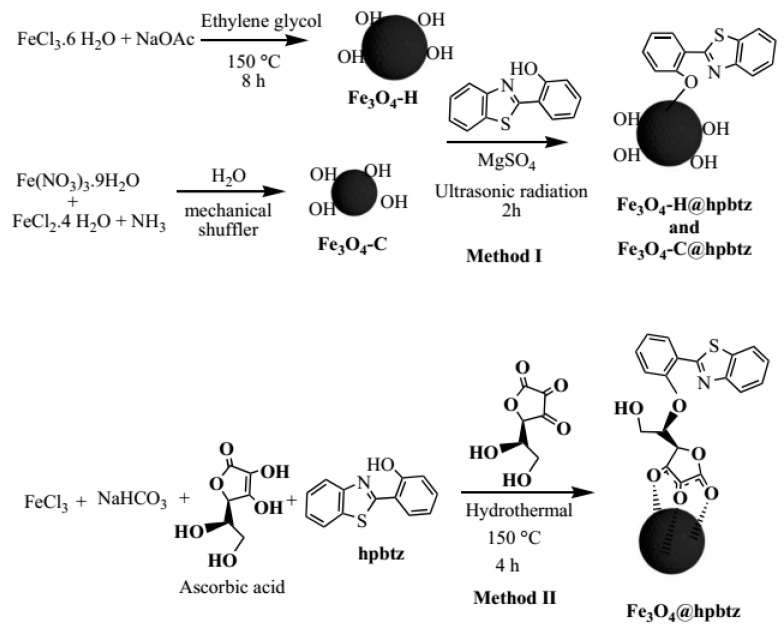
Synthesis of **hpbtz**-functionalized magnetite nanoparticles (FMNPs) by different methods.

To control the magnetite nanoparticles size, two different strategies were considered. The first pathway was the chemical reduction of Fe^3+^ using a colloidal suspension of FeCl_3_ in ethylene glycol as a reducing solvent under mild hydrothermal conditions. The second strategy was based on the chemical coprecipitation of Fe^3+^ and Fe^2+^, with a stoichiometric ratio of 2:1 (Fe^3+^/Fe^2+^) in an alkaline medium. The functionalization of the ‘as prepared’ magnetite nanoparticles, designated as Fe_3_O_4_-H and Fe_3_O_4_-C (H and C refer to the synthesis of iron oxide in hydrothermal and coprecipitation conditions, respectively), with **hpbtz** was carried out by two different but simple methods. In method (I), a straightforward approach under ultrasonic irradiation in the presence of magnesium sulfate as a water-absorbing agent was applied. On the other hand, method (II) was the preparation and functionalization of magnetite nanoparticles in a stainless-steel reactor under autogenic pressure and temperature, through a one-pot synthesis. In the latter method, colloidal [Fe(OH)_3_], was produced under mild hydrothermal conditions from FeCl_3_ and NaHCO_3_ in the ethylene glycol. Ascorbic acid (a natural compound) was used as reducing agent. Thus, the reduction of Fe^3+^ to Fe^2+^ (2:1) was regulated by the amount of ascorbic acid in the mixture. The additional hydroxyl groups provided a hydrophilic nature to the MNPs and improved their water dispersion, together with the possibility of further functionalization with the **hpbtz** ligand to produce FMNPs. The schematic presentation of the MNPs synthesis is shown in Scheme 2.

#### 2.3.1. Synthesis of Magnetite Nanoparticles under Hydrothermal Condition (Fe_3_O_4_-H)

The MNPs were synthesized with some slight modifications to the literature procedure [40]. In a typical experiment, FeCl_3_·6H_2_O (2.70 g, 10 mmol) and NaAc (7.20 g, 87.8 mmol) were dissolved in 50 mL of ethylene glycol, with stirring for 15 min. The resulting homogenous yellow solution was transferred to a Teflon-lined stainless-steel autoclave (750 mL), and then sealed and heated for 8 h in a muffle at 150 °C. The autoclave was then cooled to room temperature and the resulting black magnetite particles were centrifuged and washed with Milli-Q water (6 × 45 mL) and ethanol (2 × 10 mL). The product was dried under a vacuum at 60 °C for 12 h.

#### 2.3.2. Synthesis of Magnetite Nanoparticles by Coprecipitation (Fe_3_O_4_-C)

In a typical experiment, the Fe(NO_3_)_3_·9H_2_O (8 g, 20 mmol) and FeCl_2_.4H_2_O (2 g, 10 mmol) each were dissolved separately in water (10 mL). The two suspensions were then filtered, and the filtrates were mixed together. Subsequently, the NH_3_ in excess (8 mL, 0.34 mmol) was added drop wise while stirring with a mechanical shuffler. The mixture was maintained under vigorous stirring (1000 rpm) for 20 min. The resulting slurry was then centrifuged at 6000 rpm in cycles of 15 min. The solid product was washed with Milli-Q water (6 × 45 mL) and ethanol (2 × 10 mL), and was dried in an oven at 60 °C during 12 h.

#### 2.3.3. Preparation of Fe_3_O_4_-H@hpbtz and Fe_3_O_4_-C@hpbtz (Method I).

In a Schlenk tube, 1.2 g of Fe_3_O_4_-H NPs were added under nitrogen and were subsequently dried under a vacuum by heating at 110 °C for 24 h in order to remove the adsorbed water. **Hpbtz** (2.5 g, 10 mmol) and MgSO_4_ (2.5 g, 20 mmol) were then added to the NPs in the Schlenk tube and the mixture was dispersed in dried acetone (25 mL), using an ultrasonic bath for 2 h. The mixture was kept under stirring at room temperature for 24h. Subsequently, the reaction mixture was centrifuged at 6000 rpm in cycles of 15 min and was washed with acetone (3 × 15 mL) and Milli-Q water (3 × 45 mL) to remove the unreacted materials. The solid product (Fe_3_O_4_-H@hpbtz) was dried under a vacuum at 40 °C for 12 h. The Fe_3_O_4_-C NPs were also functionalized with an **hpbtz** ligand following the same procedure, in order to obtain Fe_3_O_4_-C@hpbtz.

#### 2.3.4. Preparation of Fe_3_O_4_@hpbtz (Method II)

The Fe_3_O_4_@hpbtz was synthesized with some slight modification to the literature procedure [41]. In a typical experiment, FeCl_3_.6H_2_O (1.35 g, 5 mmol) and NaHCO_3_(3.78 g, 45 mmol) were dissolved in 50 mL of ethylene glycol and the resulting colloidal solution was stirred with a magnetic stirrer for 15 min. Then, added to the resulting homogenous yellow solution was a solution of ascorbic acid (0.15 g, 0.8 mmol) in 10 mL ethylene glycol, with a stoichiometric ratio of 6:1, which was added to the Fe^3+^, and the mixture was stirred for 15 min. Subsequently, **hpbtz** (1.2 g, 5 mmol) was added to the reaction mixture and was then transferred to a Teflon-lined stainless-steel autoclave (750 mL), and was sealed and heated for 4 h in a muffle at 150 °C. The autoclave was then cooled to room temperature and the resulting solid was centrifuged and washed with Milli-Q water (6 × 45 mL) and ethanol (2 × 10 mL). The product was dried under a vacuum at 60 °C for 12 h.

### 2.4. Fluorescence Spectral Measurements

A 2 mg/mL stock solution of **hpbtz** functionalized MNPs and MSNs (FMNPs and FMSNs) were prepared in ethanol. The solutions of the metal cations for the fluorescence spectral analysis (Analytic-Jena, Jena, Germany) were prepared in ethanol with a concentration of 1.0 × 10^−3^ M. The excitation wavelength (λ_ex_) was set at 350 and 305 nm for FMNPs and FMSNs, respectively, with the excitation slit widths set at 5.0 nm using a quartz cell with a 1 cm optical path length. The fluorescence titration experiments were carried out by adding equal volumes (20 µL increments for FMNPs and 50 µL increments for FMSNs) of the metal ion solutions to the 2 mL samples in the quartz cell. After each addition, the solution was well mixed and kept for 2 min before the emission measurements. In order to determine the stoichiometry of the as prepared zinc complex by the Job method, the solutions of the desired functionalized materials (X) and Zn^2+^ ion were prepared with an X:Zn^2+^ ratio of 1:9, 2:8, 3:7, 4:6, 5:5, 6:4, 7:3, 8:2, and 9:1 in ethanol, keeping the total concentration = 10 µM. The emission intensity was measured at 460 nm. To avoid the precipitation of the metal ions as hydroxide, the pH was adjusted to 7.2 by the addition of HCl (5 mM) or NaOH (10 mM) in the successive experiments.

### 2.5. In Vitro Cytotoxicity Study (MTT Assay)

The human embryonic kidney (HEK293) cells were maintained in a humidified atmosphere containing 5% CO_2_ at 37 °C in Dulbecco’s Modified Eagle’s Medium (DMEM), supplemented with 1% penicillin/streptomycin, 1% sodium pyruvate, 1% glutamine, and 10% heat-inactivated fetal bovine serum (FBS). After culturing, the capacity of the FMNPs and FMSNs to interfere with the growth of HEK cells was determined with the aid of an MTT assay. The cells (1 × 10^4^ cells/well) were precultured in to 96-well microliter plates for 48 h under 5% CO_2_. The nanomaterials were added in microwells containing the cell culture, at concentrations of 50 μM. For each well, 10 μL of a MTT solution (5 mg/mL in PBS, pH = 7.4) was added and the resulting suspension was incubated for 4 h at 37 °C. After that, the medium was removed, leaving only 25 μL. The insoluble formazan was dissolved by adding 50 μL of DMSO and incubating for 10 min at 37 °C. The cell viability was determined by measuring the absorbance of each well at 540 nm, using a Bio-Rad 680 microplate reader (Bio-Rad, Hercules, CA, USA). All of the experiments were performed in triplicate. The inhibition rate (IR) compared with the control cells without nanoparticles was calculated by Equation (1), where OD was the optical density (OD), measured at 540 nm.

Inhibition rate (IR%) = [OD(control) − OD(drug treated cell)]/[OD(control)] × 100(1)

### 2.6. Fluorescence Imaging Experiments

For these experiments, the growing medium was replaced with a DMEM without phenol red. The cells were incubated in 60 mm corning plastic culture dishes (4 × 10^5^ cells/well) with the materials including the following: Fe_3_O_4_-H@hpbtz, MSN-NEt_3_-IPTMS-hpbtz-**f1**, and MSN-Pyridine-IPTMS-hpbtz-**f2** for 12 h at 37 °C and under 5% CO_2_ before imaging. Experiments to assess the Zn^2+^ uptake were performed in the same medium supplemented with 10 μM Zn^2+^ for 1 h. Before adding each nanomaterial, the supplemented medium was replaced with a normal one. Fluorescent images were captured on a Zeiss LSM 780 confocal microscope (20×, 405 nm excitation) (Zeiss, Oberkochen, Germany) and analyzed using the ImageJ and Imaris software.

### 2.7. Characterization Instruments and Conditions

The X-ray diffraction (XRD) patterns of the magnetite- and silica-based materials were recorded using a Phillips Diffractometer (Philips, Amsterdam, The Netherlands) with Cu–K*α* radiation (λ = 1.5418 Å), model PW3040/00 X’Pert MPD/MRD, at 45 KV and 40 mA. The N_2_ gas adsorption desorption isotherms were measured with a Micromeritics ASAP 2020 porosimeter (Micromeritics, Norcross, GA, USA). The pore size distributions were calculated using the Barret–Joyner–Halenda (BJH) model on the adsorption branch. The FTIR spectra of the samples were carried out using KBr pellets and were measured on a Nicolet-550FTIR spectrophotometer (Thermo Fisher Scientific Company, Waltham, MA, USA) (in the region between 400–4000 cm^−1^). The UV-vis spectra were recorded in an ethanol solution on a UV-vis Analytik Jena Specord 200PC spectrophotometer (Analytic-Jena, Jena, Germany). The S, Fe, and Si contents were determined using a semiquantitative method with an X-ray fluorescence (XRF) apparatus (Philips Magi X) (Philips, Amsterdam, The Netherlands). Transmission electron microscopy (TEM) images were taken using a Philips Tecnai 20 (Philips, Amsterdam, The Netherlands), operating at 200 kV. For the dispersion of the samples for TEM, ethanol and an ultrasonic bath were used, a drop of the ethanolic dispersed mixture was placed and evaporated on carbon-coated gold grids. The fluorescence spectra were recorded using a Perkin Elmer Infinity Plus spectrophotometer (Perkin-Elmer, Waltham, MA, USA). The cross polarization magic angle spinning ^13^C CP MAS NMR spectra were recorded on a Varian-Infinity Plus 400 MHz spectrometer (Varian Inc., Palo Alto, CA, USA), operating at 100.52 MHz proton frequency (4 µs 90° pulse, 4000 transients, spinning speed of 6 MHz, contact time 3 ms, and pulse delay of 1.5 s).

## 3. Results and Discussion

### 3.1. Synthesis and Characterization of Nanomaterials

The **hpbtz** ligand, which exhibited a significant turn-on response and a good selectivity for Zn^2+^ over potentially relevant metal ions, was prepared by a simple and eco-friendly procedure (see SI for details). It was well known that the synthesis of monodisperse particles with a uniform and homogenous size were of interest in biological applications [42,43].

Functionalized mesoporous silica nanoparticles (FMSNs) and magnetites (FMNPs) containing benzothiazole ligand (**hpbtz**) were prepared by various procedures, as depicted in Scheme 1 (silica) and Scheme 2 (magnetite).

The FMSNs were prepared using two organosilane linkers (coupling reagents), that is, 3-isocyanatopropyltriethoxysilane (ICTES) and 3-iodopropyltrimethoxysilane (IPTMS), as shown in Scheme 1. The compounds that were named as MSN-Et_3_N-NCO-hpbtz-**e1**, MSN-pyridine-NCO-hpbtz-**e2,** and MSN-NaOH-NCO-hpbtz-**e3**, were prepared by the reaction of ICTES with a hydroxyl group in **hpbtz**, to produce a covalent amide linkage (labeled as **c** in Scheme 1) in the presence of different bases with various basicities, such as triethylamine, pyridine, and sodium hydroxide, with the subsequent addition of MSNs to the resulting organosilica compounds containing an amide linkage. Moreover, the compounds, named MSN-Et_3_N-IPTMS-hpbtz-**f1**, MSN-pyridine-IPTMS-hpbtz-**f2,** and MSN-NaOH-IPTMS-hpbtz-**f3,** were prepared by the reaction of IPTMS with **hpbtz**, following the same procedure as for ICTES, except that the MSNs was added to the resulting organosilica containing ether-based compounds (labeled as **d** in Scheme 1).

In an alternative study, two series of FMNPs, named as Fe_3_O_4_-H@hpbtz and Fe_3_O_4_-C@hpbtz, were synthesized by the preparation of the MNPs under hydrothermal and co-precipitation conditions, followed by the grafting of **hpbtz** on the nanoparticle through a simple condensation process, using magnesium sulfate as a water adsorbent and under ultrasonic irradiation (Method I). In addition, an alternative procedure was used for the preparation of FMNPs that involved the synthesis of the MNPs and a subsequent reaction with **hpbtz**, in order to obtain the functionalized magnetite-based material (Fe_3_O_4_@hpbtz). In this procedure, ethylene glycol was used as a reducing solvent and ascorbic acid (vitamin C) was used as a reducing agent, which also served as capping to get NPs with exceptional solubility and stability in a water and cell culture medium. As shown in Scheme 2, the dehydroascorbic acid that was generated in situ as an oxidation byproduct acted as a stabilizing agent because of the chemical interaction of its carbonyl groups with iron oxide particles. Moreover, the exposed hydroxyl groups of dehydroascorbic acid presented the possibility of further functionalization or binding with the **hpbtz** ligand. Subsequently, the functionalization of the as prepared MNPs with **hpbtz** occurred in situ under a well-defined temperature, controlled pressure, and in one-pot synthesis (Scheme 2).

**Figure 1:**
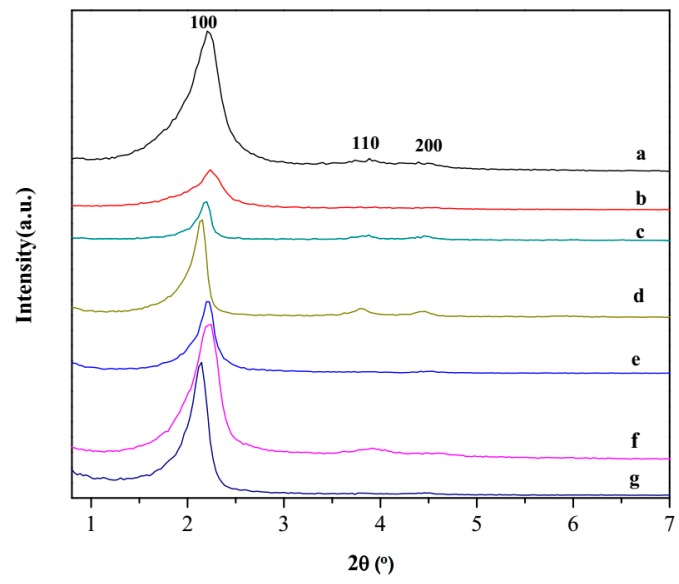
Low angle XRD patterns of (a) MSNs and FMSNs includes the following: (b) MSN-Et_3_N-IPTMS-hpbtz-**f1**, (c) MSN-pyridine-IPTMS-hpbtz-**f2**, (d) MSN-NaOH-IPTMS-hpbtz-**f3**, (e) MSN-Et_3_N-NCO-hpbtz-**e1**, (f) MSN-pyridine-NCO-hpbtz-**e2,** and (g) MSN-NaOH-NCO-hpbtz-**e3**.

The mesoscopic order of the silica-based materials was explored using X-ray diffraction (Figure 1). As expected, the low angle diffraction pattern of MSNs provided evidence of three reflection peaks at 2θ values of 1–5°, which included a high intensity peak diffracted from the (100) plane and two low intensity peaks (110) and (200), corresponding to a well-ordered one-dimensional hexagonal channels of mesoporous silica framework. The **hpbtz** containing materials, (FMSNs) in Figure 1, displayed the same pattern, demonstrating that the mesoporous structure of the MSNs scaffold was preserved after grafting the organic materials. However, the decrease in the intensity of the plane (100) diffraction peak at 2θ = 2° in FMSNs implicated the lowering of the local order, such as a variation in the wall thickness of the scaffold or reduction of the scattering contrast between the channel wall and the organic ligands that were present on the inner surface of the silica materials [44,45,46,47,48,49]. This, in turn, led to the partial lowering of the crystallinity in the FMSNs. As shown in Figure 1, MSN-Et_3_N-IPTMS-hpbtz-**f1** (with the highest incorporation of organic fractions into mesoporous silica nanoparticles scaffolds) exhibited the highest decrease in the intensity of the peaks.

**Figure 2:**
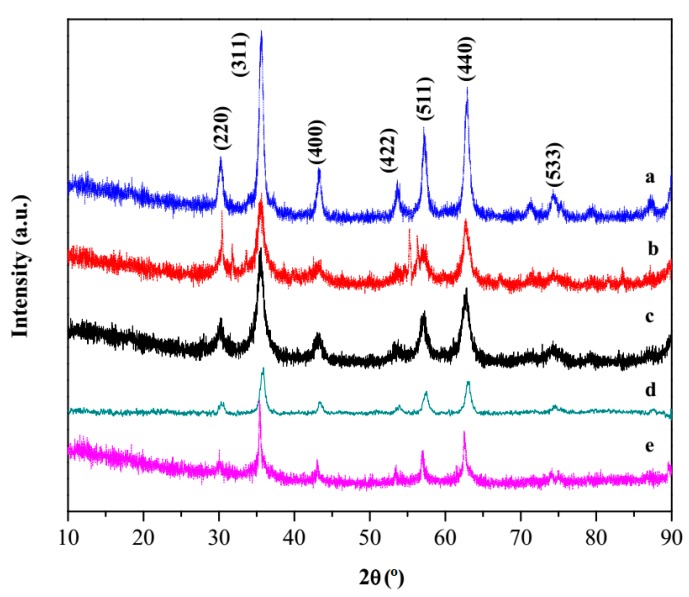
Wide angle X-Ray powder diffraction (XRD) patterns of (a) Fe_3_O_4_-H, (b) Fe_3_O_4_-C, (c) Fe_3_O_4_-H@hpbtz, (d) Fe_3_O_4_-C@hpbtz (prepared in method I), and (e) Fe_3_O_4_@hpbtz (prepared in method II).

On the other hand, in the case of magnetite-based materials, the functionalization with the **hpbtz** did not affect the X-ray diffractogram, which showed the typical peaks of magnetite in a cubic inverse spinel structure, were observed (Figure 2). The characteristic reflection peaks at 2θ values were attributed to (220), (311), (400), (422), (511), (440), and (533) planes, which agreed well with the peaks that were indexed for magnetite (JCPDS No. 89-0688), in addition, the XRD patterns were consistent with those that had been reported in the literature [50,51]. The average crystallite size, which was calculated from the broadening of the XRD peaks using Scherrer’s equation, was 33 ± 8 and 11 ± 1 nm for Fe_3_O_4_-H and Fe_3_O_4_-C, respectively [52]. To obtain the average size, the major peak (plane 311) of each product was considered. The crystallite size in Fe_3_O_4_-H was much smaller than the overall particle size of the materials (*vide infra*), presumably because of the formation of particles by several small crystallites (polycrystalline). From the diffractograms in Figure 2, it is noteworthy that the incorporation of **hpbtz** into magnetite nanoparticles did not alter the crystalline structure, as the positions of the peaks and relative intensities in the FMNPs that were prepared in methods (I) and (II) were well comparable with the pattern of the starting cubic magnetite.

**Figure 3:**
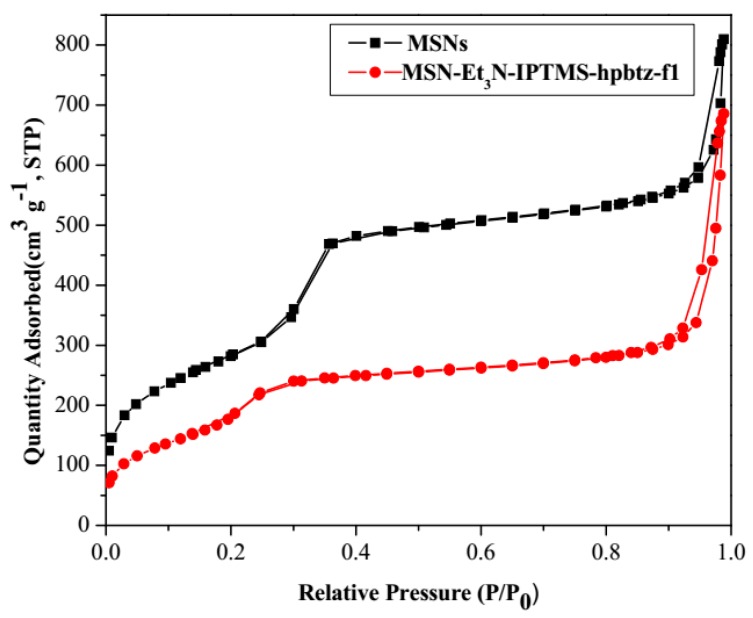
Nitrogen adsorption/desorption isotherms of MSNs and MSN-Et_3_N-IPTMS-hpbtz-**f1**. For both materials, a mixture between type IV and type VI isotherms are exhibited.

**Figure 4:**
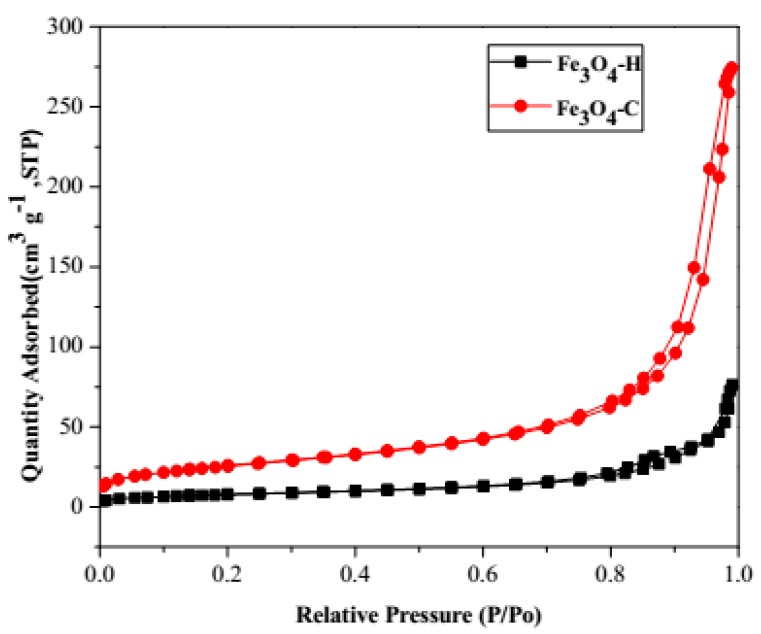
Nitrogen adsorption/desorption isotherms of MNPs (Fe_3_O_4_-H and Fe_3_O_4_-C). For both materials, type III isotherms are exhibited.

All of the materials were also characterized by nitrogen sorption. Figure 3 and Figure 4 show the nitrogen adsorption/desorption isotherms of MSNs (MSN and MSN-Et_3_N-IPTMS-hpbtz-**f1**) and MNPs (Fe_3_O_4_-H and Fe_3_O_4_-C), respectively. The physical parameters of the nitrogen isotherms, as the surface area (S_BET_), total pore volume, and BJH average pore diameter for MNPs, MSN, and **hpbtz** containing MSN are shown in Table 1.

As shown in Figure 3, the nitrogen adsorption/desorption isotherms of MSNs and MSN-Et_3_N-IPTMS-hpbtz-**f1** were between type IV and type VI isotherms, which were typical of mesoporous silica nanoparticles. In each isotherm, three different parts were differentiated; these were below 0.35, between 0.35 and 0.9, and above 0.9. The hysteresis loops in P/P_0_ = 0.8 was correlated with the existence of the capillary condensation into the mesoporous structure of MSNs, in addition, another hysteresis loop was observed at around P/P_0_ = 0.25. Physical data from Table 1 shows that MSNs material possessed a high S_BET_ of 1028.37 m^2^g^−1^ and a BJH pore diameter of 41.49 Å, with a very narrow distribution of the pore diameter. The functionalized MSNs, MSN-Et_3_N-IPTMS-hpbtz-**f1,** had a notable decrease in the S_BET_ in comparison with the initial MSN (from ca. 1000 m^2^g^−1^ to ca. 700 m^2^g^−1^), owing to the presence of IPTMS and **hpbtz** ligand inside the channels, which was also in agreement with the reduction of the pore volume from 0.97 to 0.68 cm^3^g^−1^. This phenomenon had already been found after the functionalization with different ligands [43,44,45,46,47,48,49]. Thus, our results showed that, for silica, the functionalization took place inside the pores of the system. However, in the case of the magnetite-based systems, a microporous system, the functionalization took place in the external surface area of the aggregates.

On the other hand, all of the isotherms of magnetite nanoparticles could be classified as a type III isotherm with H3 hysteresis loop, according to the IUPAC and Brunauer-Deming-Deming-Teller (BDDT) classification [53,54]. The N_2_ adsorption/desorption isotherms of Fe_3_O_4_-H and Fe_3_O_4_-C (Figure 4) exhibited the characteristic type III isotherm with a sharp increase in adsorption at P/P_0_ = 0.8–1, because of the capillary condensation of the nitrogen in the structure. Fe_3_O_4_-H and Fe_3_O_4_-C possessed a low surface area (S_BET_) of 27.96 and 92.53 m^2^g^−1^, respectively, and a BJH pore diameter of 161.69 and 175.31 Å, respectively, however, these pore diameter distributions were not narrow, which indicated the non-ordered slightly porous nature of the materials. The total pore volume and specific surface area (S_BET_) for Fe_3_O_4_-H was lower than Fe_3_O_4_-C as a result of the agglomeration of the particles under hydrothermal conditions at a sintering temperature, leading to the increase in the particle size [55].

The loading content of **hpbtz** onto the magnetite- and silica-based materials was calculated from the X-ray fluorescence (XRF) analysis, based on the sulfur content (Table 2). XRF was also used to calculate the iron and silicon contents. The sulfur content of the two FMNPs materials that were prepared by method I, Fe_3_O_4_-H@hpbtz and Fe_3_O_4_-C@hpbtz, were 0.31% and 0.54%, respectively, as compared with 0.11% for Fe_3_O_4_@hpbtz, prepared in method II. These data corresponded to 0.033, 0.096, and 0.170 mmol g^−1^ of the immobilized **hpbtz** in Fe_3_O_4_@hpbtz, Fe_3_O_4_-H@hpbtz, and Fe_3_O_4_-C@hpbtz, respectively. The sulfur and, hence, **hpbtz** loading for the FMSNs were also calculated based on the XRF analysis and the resulting data are presented in Table 2. It was found that the **hpbtz** functionalization rates were higher for the FMSNs that were prepared in the post functionalization reaction with IPTMS, and the amount of immobilized **hpbtz** increased in the IPTMS that contained materials as 0.016, 0.018, and 0.056 mmol g^−1^ for MSN-pyridine-IPTMS-hpbtz-**f2,** MSN-NaOH-IPTMS-hpbtz-**f3,** and MSN-Et_3_N-IPTMS-hpbtz-**f1**, respectively.

In addition, the thermal behavior of silica- and magnetite-based materials were investigated using a thermogravimetric analysis (Figures S1 and S2 of Supplementary Material), and the resulting data are given in Table 3.

The thermogravimetric analysis of MSNs and MSN-NEt_3_-IPTMS-hpbtz-**f1** (Figure S1) showed an initial weight loss of up to 190 °C, assigned to the desorption of the residual water molecules that were trapped in the silica porous structure. The next main weight loss at above 200 °C was probably because of the decomposition of the organic fractions. It seemed that the main weight loss, with about 1.44% in the thermogram of the MSNs, originated from the condensation of the silanol groups of the material, as it was mainly observed above 550 °C. Moreover, the amount of the immobilized organic fractions onto MSN-NEt_3_-IPTMS-hpbtz-**f1** was estimated to be 18.54%, corresponding to the presence of IPTMS and the **hpbtz** ligand.

On the other hand, in the thermograms of the magnetite-based materials (Figure S2), a weight loss between around 190 to 450 °C of between ca. 1.2% and 8.7% (Table 3) was attributed to the removal of organic moieties (including ethylene glycol, ascorbic acid, and **hpbtz**) on the nanoparticles surface. As it was clear from the thermal analysis data, Fe_3_O_4_-C@hpbtz had the highest weight loss compared with the other magnetite-based materials because of its higher organic content. Indeed, the thermogram of Fe_3_O_4_-C@hpbtz showed a weight loss between 190 and 450 °C, including three steps, presumably because of the decomposition of different organic fractions that were composed of ascorbic acid, as stabilizing agent; **hpbtz** ligand; and residual ethylene glycol grafted on the nanoparticles surface.

**Figure 5:**
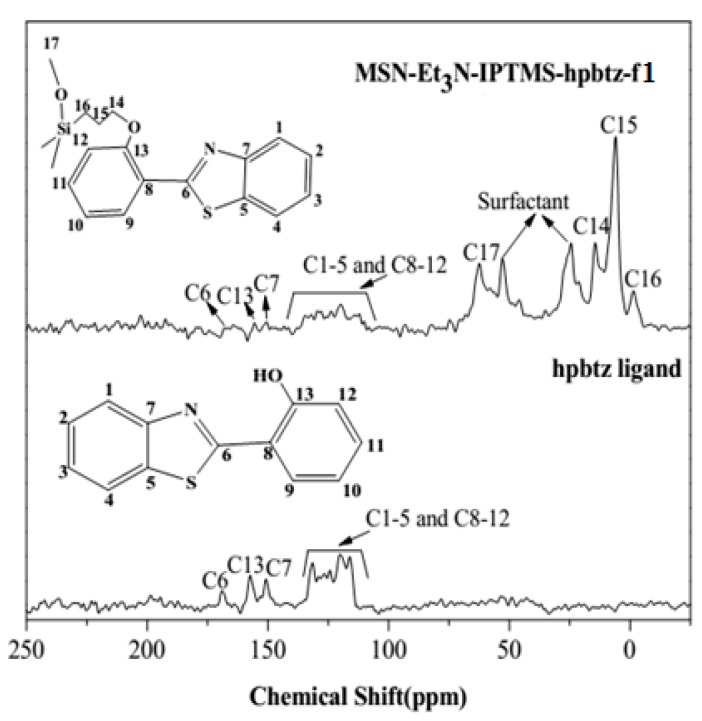
^13^C CP-MAS NMR spectra of **hpbtz** ligand and MSN-Et_3_N-IPTMS-hpbtz-**f1**.

The ^13^C CP-MAS NMR spectra were performed to elucidate the presence of **hpbtz** in the silica-based materials. Figure 5 represents the solid state ^13^C CP-MAS NMR spectra of **hpbtz** and MSN-Et_3_N-IPTMS-hpbtz-**f1**. The **hpbtz** spectrum exhibited three signals for C6=N, C13-O, and C7-N at 166.6, 155.3, and 154.4 ppm, respectively. The remaining phenyl carbons (C1-5 and C8-12) of **hpbtz** appeared in the 115 to 140 ppm region. In the spectrum of MSN-Et_3_N-IPTMS-hpbtz-**f**, the three signals at 26.0, 22.6, and 14.1 ppm corresponded to C14, C15, and C16, respectively, of the methylene carbon atoms of the silylating agent (IMTPS). In addition, an intense signal at 63.0 ppm was characteristic of the methoxy carbon atom of the IPTMS. The ^13^C MAS NMR spectrum of MSN-Et_3_N-IPTMS-hpbtz-**f1** showed the corresponding signals for the carbon atoms of **hpbtz** with low intensity and in approximately the same chemical shift range (116.4 to 166.6 ppm). The results confirmed that **hpbtz** was grafted into the MSNs cavities following the post-functionalization process with IPTMS. The FTIR and UV-vis spectral analyses gave further support to these observations (for details: see Supporting Materials, Figures S3–S6).

**Figure 6:**
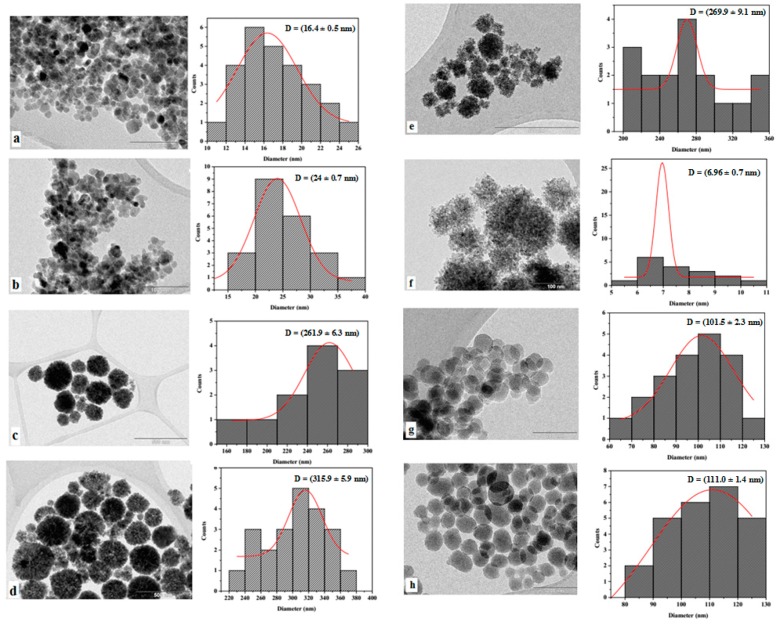
TEM analysis of MNPs and FMNPs for (**a**) Fe_3_O_4_-C, (**b**) Fe_3_O_4_-C@hpbtz, (**c**) Fe_3_O_4_-H, (**d**) Fe_3_O_4_-H@hpbtz, (**e**) Fe_3_O_4_@hpbtz, (**f**) Fe_3_O_4_-H, (**g**) MSNs, and (**h**) MSN-Et_3_N-IPTMS-hpbtz-**f1,** as well as the corresponding particle size distribution.

In order to determine the structure and size distribution of the synthesized materials, the magnetite- and silica-based particles were characterized by TEM (Figure 6). Figure 6 shows the TEM images of Fe_3_O_4_-C (a), Fe_3_O_4_-C@hpbtz (b), Fe_3_O_4_-H (c), and Fe_3_O_4_-H@hpbtz (d) that were prepared by hydrothermal and coprecipitation methods. As evident from Figure 6, the MNPs that were prepared by coprecipitation method and its functionalized counterpart (a and b) had a “quasi”-spherical morphology with a small degree of agglomeration. As calculated by the ImageJ software, a and b had a narrow particle size distribution with average diameters of about 16.4 ± 0.5 and 24.0 ± 0.7 nm, respectively. However, the MNPs that were prepared in the hydrothermal method and its functionalized counterpart (c and d) seemed to be more regular, they were forming spherical aggregates (of ca. 200–300 nm) of very small nanoparticles (of ca. 10–30 nm). It seemed that every particle was formed by small NPs that had accumulated in porous aggregates. The estimated size distribution for the MNPs that were synthesized under the hydrothermal condition were 261.9 ± 6.3 and 315.9 ± 5.9 nm for c and d, respectively, and the value was higher than the materials that were prepared in the coprecipitation method. In addition, a size distribution of about 269.9 ± 9.1 was obtained for the magnetite functionalized NPs, which were prepared in a one-pot hydrothermal procedure (method II) (Figure 6e). The growth of very small nanoparticles into the porous structure during one-pot hydrothermal synthesis was apparent in Figure 6f.

The TEM image of the MSNs (Figure 6g) revealed the mesoporous structure and ordered arrangements of the pores with a size distribution of 101.5 ± 2.3 nm. As evident from Figure 6h for MSN-Et_3_N-IPTMS-hpbtz-**f1**, the original morphology, arrangement, and pore distribution of the mesoporous materials were maintained during the functionalization with the organic compounds. The estimated size distribution for h was about 111.0 ± 1.4 nm and showed a small increase in the size after functionalization with organic moieties, which was only probably because of a statistic factor.

### 3.2. UV–Vis Titration

**Figure 7:**
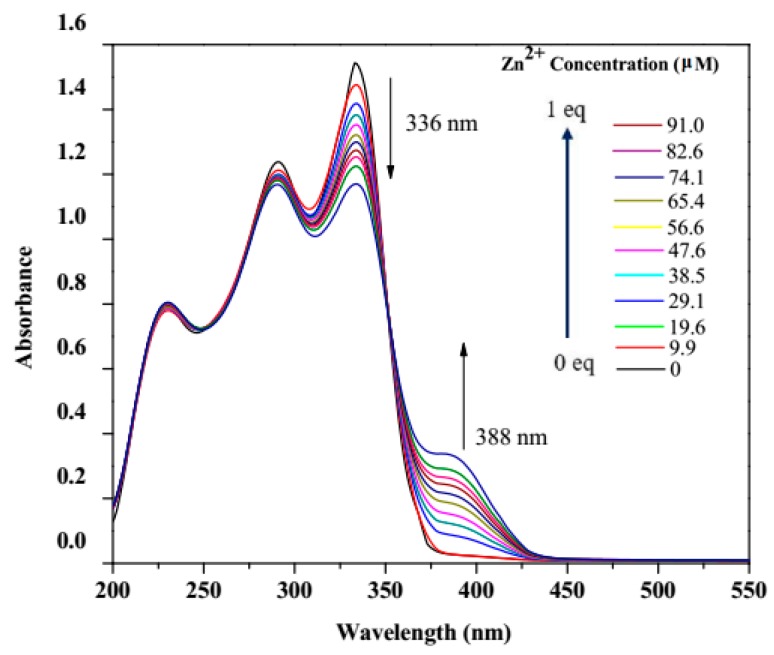
UV-vis titration of an ethanolic solution of **hpbtz** (1 × 10^−4^ M) with Zn^2+^ ion (1 × 10^−3^ M).

To investigate the electronic absorption and subsequent emission properties of the systems, the electronic spectrum of **hpbtz** in ethanol was recorded and showed three peaks at 219, 288, and 336 nm (Figure 7). The **hpbtz**-Zn^2+^ complexation was monitored by the titration experiment. The study was based on the modification of the UV-vis absorption spectra of **hpbtz** (1 × 10^−4^ M), with various concentrations of Zn^2+^ ion (1 × 10^−3^ M). After the complexation with Zn, the absorbance of the peaks of the ligand modified and there appeared to be an isosbestic point at 352 nm, which was in agreement with the zinc complexation. Additionally, a new band was at 388 nm, because the charge transfer transition increased in intensity with increasing amounts of zinc ion.

### 3.3. Fluorescence Study

**Figure 8:**
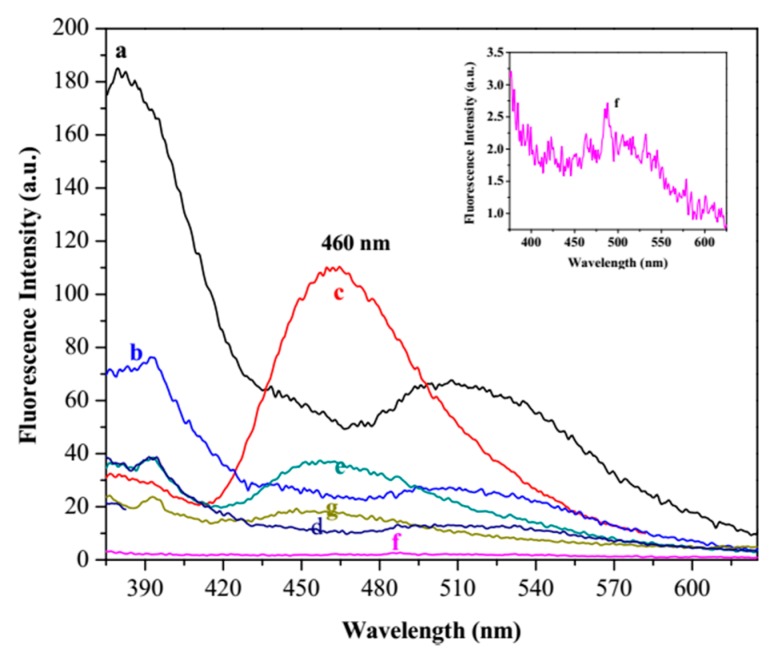
Comparative fluorescence spectra of (a) **hpbtz**, (b) Fe_3_O_4_-H@hpbtz, (c) Fe_3_O_4_-H@hpbtz + Zn^2+^, (d) Fe_3_O_4_-C@hpbtz, (e) Fe_3_O_4_-C@hpbtz + Zn^2+^, (f) Fe_3_O_4_@hpbtz, and (g) Fe_3_O_4_@hpbtz + Zn^2+^, upon the addition of Zn^2+^ (7.8 equivalent, 300 μL), at a concentration of 10^−3^ M in an ethanol solution (λ_ex_ = 350 nm) (Inset: **f** in higher concentration, 3 mg mL^−1^).

**Figure 9:**
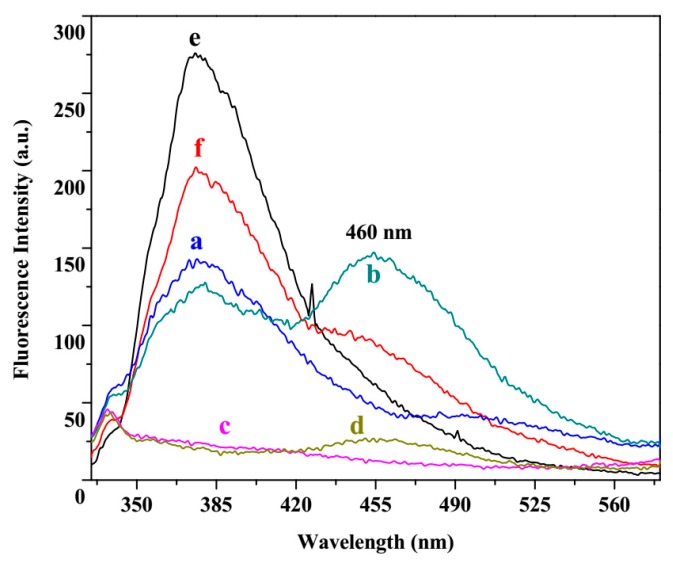
Comparative fluorescence spectra of (a) MSN-Et_3_N-IPTMS-hpbtz-**f1**, (b) MSN-Et_3_N-IPTMS-hpbtz-**f1** + Zn^2+^, (c) MSN-pyridine-IPTMS-hpbtz-**f2**, (d) MSN-pyridine-IPTMS-hpbtz-**f2** + Zn^2+^, (e) MSN-NaOH-IPTMS-hpbtz-**f3**, and (f) MSN-NaOH-IPTMS-hpbtz-**f3** + Zn^2+^, upon the addition of Zn^2+^ (15.4 equivalent, 350 μL) at a concentration of 10^−3^ M in ethanol (λ_ex_ = 305 nm).

To investigate the emission properties of the synthesized materials for zinc detection in a solution, functionalized magnetite- and silica-based nanoparticles were studied by monitoring the changes in the fluorescence emission intensity upon the addition of 300 µL (7.8 equivalent) of Zn^2+^, at a concentration of 10^−3^ M, to 2 mL of the ethanolic suspension of FMNPs (2 mg mL^−1^), and 350 µL (15.4 equivalent) of the same Zn^2+^ solution to (2 mg mL^−1^) of the ethanolic suspension of FMSNs, following the excitation at 305 nm (Figure 8 and Figure 9). The comparative fluorescence spectra of the functionalized magnetite-based materials (FMNPs) in the presence of Zn^2+^ indicated a new band with a maximum located at around 460 nm (Figure 8). This new band, which was induced by Zn^2+^, was more intense in c (Fe_3_O_4_-H@hpbtz) compared with e and g, and was attributed to the complexation of the grafted **hpbtz** ligand in Fe_3_O_4_-H@hpbtz with zinc ion.

In addition, Figure 9 presents the comparative fluorescence spectra of FMSNs, MSN-Et_3_N-IPTMS-hpbtz-**f1**, MSN-pyridine-IPTMS-hpbtz-**f2**, and MSN-NaOH-IPTMS-hpbtz-**f3**, upon addition of 350 µL (15.4 equivalent) of Zn^2+^ solution in ethanol (10^−3^ M). As demonstrated in Figure 9, all three of the silica-based materials showed a new emission peak at around 460 nm, in the presence of Zn^2+^.

The new emission band at around 460 nm, with an isoemission point at 430 nm, was presumably related to the complexation of the Zn^2+^ ion by the grafted **hpbtz** in the silica-based materials. It seemed that the higher fluorescence intensity that was observed in MSN-Et_3_N-IPTMS-hpbtz-**f1** was related to the higher content of grafted **hpbtz** and hence, the higher Zn^2+^ ion complexation.

Since the loaded **hpbtz** onto the nanoparticles was different (as proved by an X-ray fluorescence spectroscopy), the Fe_3_O_4_-H@hpbtz and MSN-Et_3_N-IPTMS-hpbtz-**f1** dispersions with the highest **hpbtz** loads were selected for investigating the probe activity of the synthesized materials in the fluorescence titration and binding studies with Zn^2+^ ion.

### 3.4. Fluorescence Titration and Binding Studies

**Figure 10:**
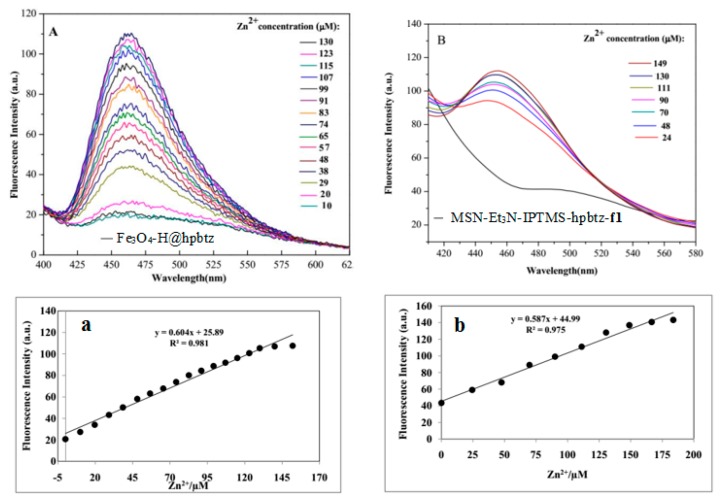
Fluorescence intensity changes observed in (**A**) Fe_3_O_4_-H@hpbtz (2 mg mL^−1^) and (**B**) MSN-Et_3_N-IPTMS-hpbtz-**f1** (2 mg mL^−1^) upon the addition of Zn^2+^ (0–7.8 equivalent, 0–130 μM and 300 μL for A, and 0–15.4 equivalents, 0–130 μM and 350 μL for B) at λ_ex_ = 350 nm for Aa and 305 nm for B in the ethanol (a and b: plot of the fluorescence intensity at 460 nm as a function of the Zn^2+^ concentration.

To determine the zinc binding ability of Fe_3_O_4_-H@hpbtz and MSN-Et_3_N-IPTMS-btz-**f1,** a fluorescence titration with an increasing concentration of Zn^2+^ ions was carried out (Figure 10A,B). The fluorescence emission spectra were recorded when the different concentrations of Zn^2+^ were added to the ethanolic dispersion of magnetite- and silica-based materials. Through the gradual increase of the Zn^2+^ concentration, the new emission band at λ = 460 nm in Fe_3_O_4_-H@hpbtz and MSN-Et_3_N-IPTMS-btz-**f1** increased and reached the highest value after the addition of 7.8 and 15.4 equivalent of Zn^2+^ solution, as depicted in 10 a and b, respectively. From a and b in Figure 10, which present the plot of the fluorescence intensity versus [Zn^2+^], based on the fluorescence titration data, a good linearity between the fluorescence intensity and Zn^2+^concentration (0–7.8 equivalent, 0–130 μM for Fe_3_O_4_-H@hpbtz and 0–15.4 equivalent, 0–149 µM for MSN-Et_3_N-IPTMS-hpbtz-**f1**) was observed, with a regression coefficient, R^2^, with values of 0.981 and 0.975, respectively.

**Figure 11:**
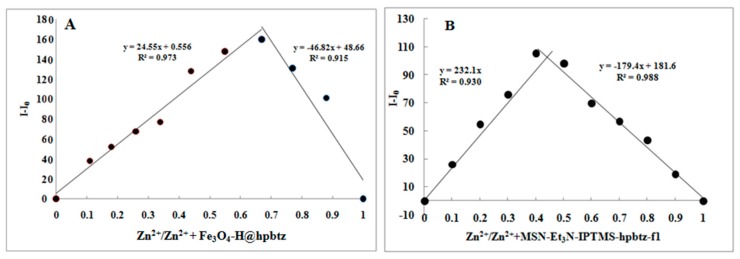
Job’s plot indicating the 2:1 stoichiometry for [Zn^2+^: Fe_3_O_4_-H@hpbtz] (**A**) and 1:2 for [Zn^2+^: MSN-Et_3_N-IPTMS-hpbtz-**f1**] (**B**). The total concentration of (L) and Zn^2+^ is 10 µM (λ_ex_ = 350 and 305 for Fe_3_O_4_-H@hpbtz and MSN-Et_3_N-IPTMS-hpbtz-**f1**, respectively).

To determine the binding stoichiometry of Zn^2+^ to Fe_3_O_4_-H@hpbtz and MSN-Et_3_N-IPTMS-hpbtz-**f1**, Job’s plot analysis was applied [56,57]. In Figure 11A,B, the emission intensity at 460 nm was plotted against the mole fraction of Fe_3_O_4_-H@hpbtz and MSN-Et_3_N-IPTMS-hpbtz-**f1**, respectively, at a constant total concentration (10 µM). The maximum emission intensity was reached at the mole fraction of around 0.7 in A and 0.4 in B, indicating a 1:2 ratio [L:M] in a and 2:1 [L:M] in b [58,59].

**Figure 12:**
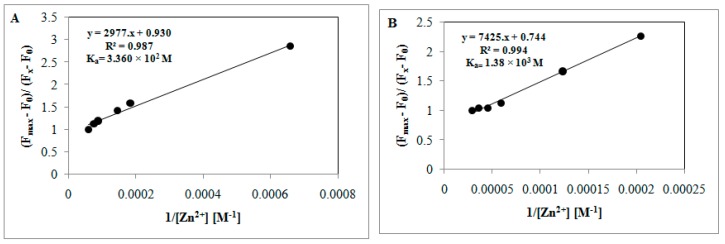
The association constant (K_a_) of each sensor with Zn^2+^ was calculated by Benesi–Hildebrand equation for Fe_3_O_4_-H@hpbtz (**A**) and MSN-Et_3_N-IPTMS-hpbtz-**f1** (**B**).

Moreover, the Zn^2+^ binding affinity of magnetite- and silica-functionalized materials was further confirmed by calculating the association constant (K_a_), using the Benesi–Hildebrand Equation (2) (Figure 12A,B). F_0_, F_x_, and F_max_, were the emission intensities of two nano-sensors in the absence of Zn^2+^, in an intermediate Zn^2+^ concentration, and at a concentration of Zn^2+^ with complete interaction, respectively. Based on the Job’s plot results, the n value in Equation (1) was calculated as n = 2 for Fe_3_O_4_-H@hpbtz and n = 1 for MSN-Et_3_N-IPTMS-hpbtz-**f1**.

F_max_ − F_0_/F_x_ − F_0_ = 1 + (1/K [M]^n^) (2)

The calculated association constant for Fe_3_O_4_-H@hpbtz and MSN-Et_3_N-IPTMS-hpbtz-**f1** was found to be 3.360 × 10^2^ and 1.38 × 10^3^ M, respectively. The detection limit was also calculated based on Equation (3), as follows:

DL = KS_d_/m (3)

Where K is generally set to 3, S_d_ is the standard deviation of the blank solution, and m is the slope of fluorescence intensity versus the Zn^2+^ concentration. The detection limits were calculated to be 2.53 × 10^−6^ and 2.55 × 10^−6^ M for Fe_3_O_4_-H@hpbtz and MSN-Et_3_N-IPTMS-hpbtz-**f1**, respectively.

**Figure 13:**
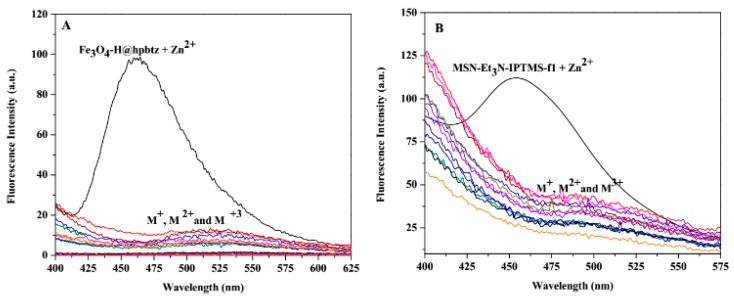
Fluorescence emission spectra for (**A**) Fe_3_O_4_-H@hpbtz in the presence of 7.8 equivalent, 300 μL and (**B**) MSN-Et_3_N-IPTMS-hpbtz-**f1** in the presence of 15.4 equivalent, 350 μL of various metal ions at λ = 350 and 305 nm, respectively, in ethanol.

To study the effect of the metal ions, the binding behavior of the Fe_3_O_4_-H@hpbtz and MSN-Et_3_N-IPTMS-hpbtz-**f1** to different metal cations as their nitrate salts, were monitored using fluorescence titration. All of the titrations were carried out in ethanolic solutions at pH = 7.2 (Figure 13A,B).

Different metal cations, including Na^+^, K^+^, Ag^+^, Ca^2+^, Cu^2+^, Zn^2+^, Co^2+^, Ba^2+^, Ni^2+^, Mg^2+^, Pb^2+^, Fe^2+^, Al^3+^, Cd^2+^, and Hg^2+^ were tested. The emission spectrum of Fe_3_O_4_-H@hpbtz in Figure 13A shows an emission enhancement with the addition of Zn^2+^ (7.8 equivalent, 130 µM, 300 µL) at 460 nm. Other transition and alkali metals did not show any significant effect on the fluorescence intensity of Fe_3_O_4_-H@hpbtz under the same experimental conditions. Also, in Figure 13B, the emission spectrum of MSN-Et_3_N-IPTMS-hpbtz-**f1** showed a fluorescence emission band at 460 nm with a significant enhancement under a steady increase of Zn^2+^ concentration (0–15.4 equivalent, 0–149 µM, 350 μL). Moreover, other transition and alkali metals did not show any significant effect on the fluorescence intensity of MSN-Et_3_N-IPTMS-hpbtz-**f1** under the same experimental conditions. The observed high fluorescent intensity was most probably attributed to the formation of a rigid system after the **hpbtz** fluorophore binding to Zn^2+^, leading to the chelation-enhanced fluorescence effect (CHEF) [60,61].

**Figure 14:**
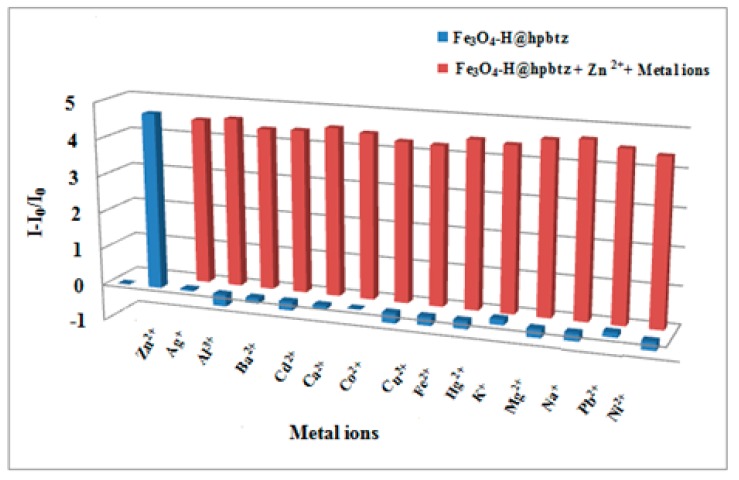
Variation of the fluorescence intensity of Fe_3_O_4_-H@hpbtz (2 mg mL^−1^) with Zn^2+^ (7.8 equivalent, 300 μL) and various metal ions (2 times more in equivalent) at 460 nm (λ_ex_= 350 nm) in ethanol.

**Figure 15:**
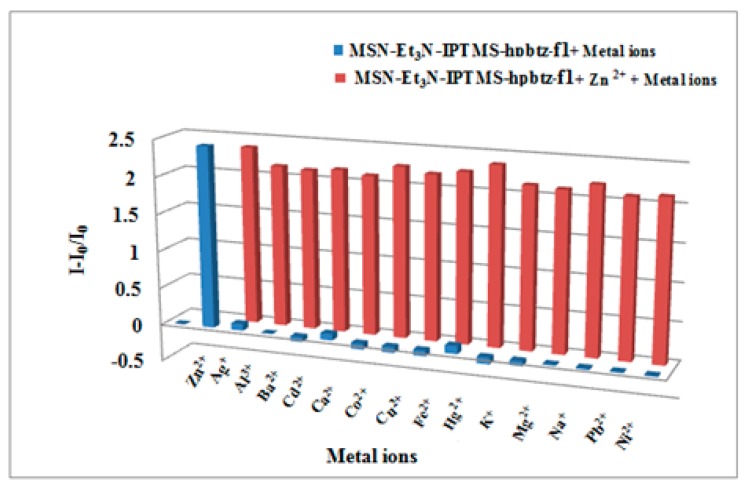
Variation of the fluorescence intensity of MSN-Et_3_N-IPTMS-hpbtz-**f1** (2 mg mL^−1^), with Zn^2+^ (15.4 equivalent, 350 μL) and various metal ions (two times more in equivalent) at 460 nm (λ_ex_= 305 nm) in ethanol.

Furthermore, the selectivity of Fe_3_O_4_-H@hpbtz and MSN-Et_3_N-IPTMS-hpbtz-f1toward Zn^2+^ ion was studied as shown in Figure 14 and Figure 15, as one of the most crucial features of a sensor in the competition experiments was its selectivity toward a target ion. Therefore, the possible interference of Ag^+^, Al^3+^, Ba^2+^, Cd^2+^, Ca^2+^, Co^2+^, Cu^2+^, Fe^2+^, Hg^2+^, K^+^, Mg^2+^, Na^+^, Pb^2+^, and Ni^2+^ on the fluorescence behavior of Fe_3_O_4_-H@hpbtz and MSN-Et_3_N-IPTMS-hpbtz-**f1** was examined. In these experiments, the competitive metal ions were added in excess (2 equivalent) to a solution of Fe_3_O_4_-H@hpbtz and MSN-Et_3_N-IPTMS-hpbtz-**f1** in the presence of Zn^2+^. As depicted in Figure 14 and Figure 15, no significant changes were observed in the fluorescence response of both magnetite- and silica-based probes for Zn^2+,^ in the presence of other competitive metal ions at 460 nm. Notably, the sensitivity of Fe_3_O_4_-H@hpbtz and MSN-Et_3_N-IPTMS-hpbtz-**f1** for Zn^2+^ did not have any interference from Cd^2+,^ even though Cd^2+^and Zn^2+^ had similar chemical properties and generally caused a strong interference when they were present together in a solution [62,63].

**Scheme 3:**
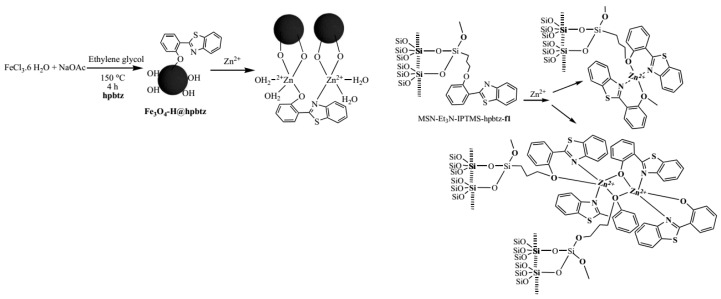
Proposed binding mechanism of Fe_3_O_4_-H@hpbtz or MSN-Et_3_N-IPTMS-hpbtz-**f1** and Zn^2+.^

These results confirmed that both Fe_3_O_4_-H@hpbtz and MSN-Et_3_N-IPTMS-hpbtz-**f1** could be used as selective fluorogenic probes with a high affinity for Zn^2+^, in comparison to the other competitive metal ions. Accordingly, the proposed binding mechanisms for the coordination of Fe_3_O_4_-H@hpbtz and MSN-Et_3_N-IPTMS-hpbtz-**f1** to Zn^2+^ are shown in Scheme 3, and the proposed species were based on the X-ray crystal structure of [Zn(BTZ)_2_]_2_, which was reported previously [59].

### 3.5. Influence of FMNPs and FMSNs on Cell Viability of HEK293 Cell Line

**Figure 16:**
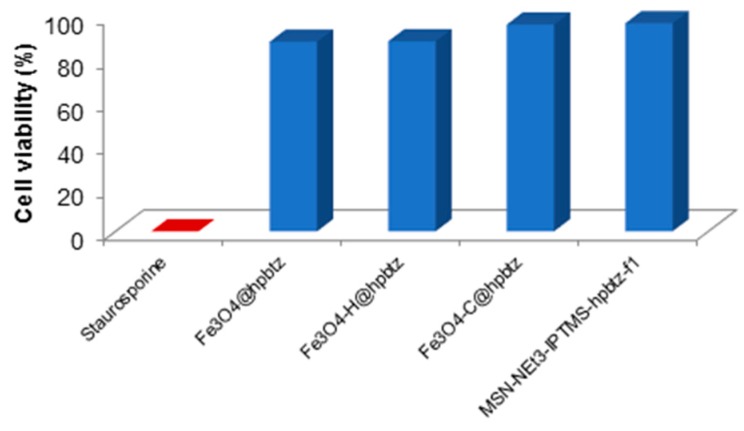
Toxicity of tested nanomaterials on HEK293 cells.

As the MTT assay evaluated the cell viability, this assay was selected to assess whether the synthesized magnetite- and silica-based materials (Fe_3_O_4_-H@hpbtz, Fe_3_O_4_-C@hpbtz, Fe_3_O_4_@hpbtz, and MSN-NEt_3_-IPTMS-hpbtz-**f1)** should have had an impact on this crucial parameter using a HEK293 cell line [64]. Toward this scope, HEK293 cells were incubated with 50 μM of the materials for 48 h at 37 °C in the 5% CO_2_ atmosphere. These results, which were reported as the inhibition rate (%) (Figure 16), showed negligible cellular toxicity for both magnetite- and silica-based NPs. In fact, in all of the cases, the inhibition rate was always below 20% (about 16.27% for Fe_3_O_4_-H@hpbtz, 16.47% for Fe_3_O_4_@hpbtz, 8.45% for Fe_3_O_4_-C@hpbtz, and 7.8% for MSN-NEt_3_-IPTMS-hpbtz-**f1**). The very low cell toxicity was an interesting feature for the synthesized materials and provided the possibility of being used as potential imaging agents. Interestingly, the higher the NPs size, the lower the cell viability. Furthermore, the inhibition rate of the magnetite-based materials was always higher than that of the silica nanoparticles. In fact, within the three functionalized MNPs with different particle size distribution, that is, Fe_3_O_4_-H@hpbtz (315 nm), Fe_3_O_4_-C@hpbtz (FMNPs prepared by method I, ca. 260 nm), and Fe_3_O_4_@hpbtz (FMNPs prepared by method II, 270 nm), Fe_3_O_4_-C@hpbtz (24 nm) had the minimum toxicity effect.

On the other hand, MSN-NEt_3_-IPTMS-hpbtz-**f1** with a particle size distribution of 111 nm and a well-ordered porous structure had a lower toxicity, relative to the three FMNPs, and hence more cell viability compared to the magnetite-based materials.

### 3.6. Live-Cell Imaging

The ability to uptake Zn^2+^ ions using synthesized FMNPs and FMSNs in HEK293 cells was studied by CLSM. Prior to the analysis, Fe_3_O_4_-H@hpbtz, MSN-NEt_3_-IPTMS-hpbtz-**f1**, and MSN-pyridine-IPTMS-hpbtz-**f2** were incubated with HEK293 cells, without external supplementing of Zn^2+^ ions and, as expected, the cells showed no significant fluorescence signal upon incubation.

**Figure 17:**
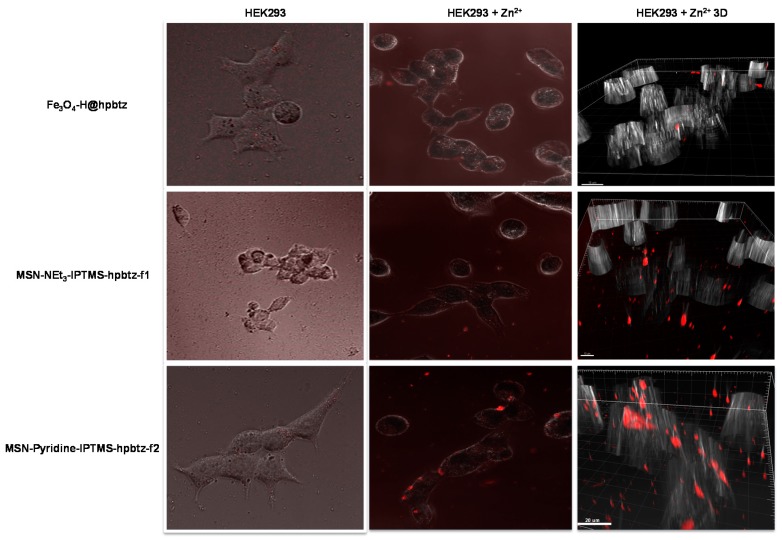
Fluorescence images of the HEK293 cells treated with different functionalized materials in absence of Zn^2+^ supplementation (Column I), in the presence of supplemented Zn^2+^ (Column II) and 3D reconstruction of cells treated with nanomaterials in the presence of Zn^2+^ (Column III).

After supplementing the culture growing medium with Zn^2+^, washing, and their incubation, the nanoparticles became fluorescent, appearing as red spots (Figure 17). For example, the cells that were grown in the presence of Zn^2+^ and were treated with Fe_3_O_4_-H@hpbtz, showed these red spots, which were indicating agglomeration of magnetite nanoparticles. The 3D reconstruction confirmed the same evidence. Apparently, most of the magnetite-based nanoparticles were located outside (Figure 17). In the case of Fe_3_O_4_-H@hpbtz, the nanoparticles internalization was found to be lower, enabling a very poor contrast for confocal imaging. It was supposed that the inherent tendency of magnetite nanoparticles for agglomeration and hence particle growth, hindered their efficient internalization into the cells and cellular uptake of zinc ions [65,66].

Interestingly, the silica-based materials gave stronger fluorescence compared with the magnetite-based nanoparticles (Figure 17). Even the 3D reconstruction of the HEK293 cells that were supplemented in Zinc ions and were treated with the aforementioned nanoparticles confirmed these results. This evidence demonstrated a higher cell membrane-permeation of mesoporous silica nanoparticles and, therefore, a higher internalization of the silica particles. This behavior made them good candidates for being used as optical imaging probes to detect the pathological dysregulation of intracellular Zn^2+^ in living cells. Indeed, it was well known that Zn^2+^ played an important role as a cellular messenger in physiological and cytotoxic signaling in neuronal cell death [67]. In addition, the possibility of detecting Zn^2+^ ions in the central nerve system could help in the tracking of brain development to detect either low concentrations, which may lead to apoptosis of the brain cells [68], or high Zn^2+^ concentrations, which may have led to necrosis of the brain cells [69]. In addition, these materials, with an optimization of their internalization capacity and intensity for intracellular Zn^2+^ signaling could be of high interest for determining the action of Zn^2+^ in ischemia-induced injuries [70].

## 4. Conclusions

New functionalized magnetite and silica nanoparticles (FMNPs and FMSNs) have been synthesized through different methods and have been functionalized using a benzothiazole-based fluorophore ligand via several procedures. For the synthesis of FMNPs, two procedures, including coprecipitation under ultrasonic irradiation and one-pot synthesis, were applied. In the second methodology, ascorbic acid was used as a reducing and capping agent to produce an oxidation byproduct as a stabilizer, under hydrothermal conditions. Additionally, FMSNs have been prepared through a post-functionalization method (PSM), using two organosilane coupling agents, IPTMS and ICTES, in the presence of three bases with different strengths for the deprotonation of **hpbtz** and the subsequent reaction with the nanostructured materials. The covalent attachment of the **hpbtz** makes the FMNPs system more stable. Diverse synthetic methods and **hpbtz** loading conditions led to different particle size distribution and ligand immobilization. Among the synthesized materials, the functionalized magnetite nanoparticles prepared in method (I), Fe_3_O_4_-H@hpbtz, and the functionalized mesoporous silica prepared through post functionalization with the IPTMS coupling agent, had a high extent of **hpbtz** ligand and, because of their good photophysical properties, were applied as zinc ion probes in ethanol. Both probes showed selective and sensitive off-on responses toward Zn^2+^. In addition, the MTT assay of the magnetite- and silica-based materials on the HEK293 cell line and the utilization of the new synthesized probes in cell imaging for Zn^2+^ cellular uptake has been demonstrated. The results indicated that magnetite-based nanoparticles functionalized with Zn-selective ligands (hpbtz) were compatible with cells (toxicity lower than 20%). However, despite the low toxicity for being used as imaging agents, the ligand functionalization was low and the intensity of the cell signaling was not optimal, limiting their applicability. Furthermore, the internalization of the nanoparticles inside the cells was moderate, therefore, a targeting molecule was needed to improve cell uptake. On the other hand, the silica-based nanoparticles containing **hpbtz** were not toxic to cells (lower than 8%), indicating their potential applicability as in vitro imaging agents. Despite having a relatively low functionalization with **hpbtz**, the intensity of the cell signaling was very high and was easily observed through in vitro study. Compared with other similar detection systems that have been described in the literature, the nanoparticles that were reported here showed a higher sensibility for being able to detect the target metal in the cells that were supplemented with only 10 µM Zn^2+^. Conversely, recent studies with other nanoparticles or organic compounds described lower sensibility, showing a higher detection limit of Zn^2+^ concentration, for example 20 µM [71], 25 µM [72], or even 10 mM [73].

Finally, it is worth noting that silica-based nanoparticles do not need a targeting molecule to internalize cells, which is an interesting result that enhances the capacity of these materials to be used as in vitro signaling agents for detecting excess of Zn^2+^ in cells.

## Figures and Tables

**Table 1:** Textural properties of magnetite nanoparticles (MNPs), mesoporous silica nanoparticles (MSNs), and MSN-Et_3_N-IPMS-hpbtz-**f1**.

Material	S_BET_ (m^2^ g^−1^)	Pore Volume (cm^3^ g^−1^)	Pore Size (Å)
Fe_3_O_4_-H	27.96	0.072	161.69
Fe_3_O_4_-C	92.53	0.319	175.31
MSNs	1028.37	0.970	41.49
MSN-Et_3_N-IPTMS-hpbtz-**f1**	665.91	0.682	57.20

**Table 2:** Composition of synthesized materials determined by XRF.

Material	Fe, Si Content (%)	S (%)	hpbtz (m mol g^−1^)
Fe_3_O_4_-H	Fe (61.4)	-	-
Fe_3_O_4_-C	Fe (74.2)	-	-
Fe_3_O_4_-H@hpbtz	Fe (75.2)	0.307	0.096
Fe_3_O_4_-C@hpbtz	Fe (60.3)	0.543	0.170
Fe_3_O_4_@hpbtz	Fe (64.5)	0.107	0.033
MSNs	Si (22.7)	-	-
MSN-Et_3_N-IPTMS-hpbtz-**f1**	Si (28)	0.178	0.056
MSN-pyridine-IPTMS-hpbtz-**f2**	Si (19.0)	0.051	0.016
MSN-NaOH-IPTMS-hpbtz-**f3**	Si (17.0)	0.057	0.018
MSN-Et_3_N-NCO-hpbtz-**e1**	Si (9.70)	0.010	0.003
MSN-pyridine-NCO-hpbtz-**e2**	Si (11,6)	0.025	0.008
MSN-NaOH-NCO-hpbtz-**e3**	Si (8.97)	0.021	0.006

**Table 3:** Weight loss of the synthesized materials in thermogravimetry experiments.

Material	% Weight Loss (20–190 °C)	% Weight Loss (190–450 °C)
Fe_3_O_4_-H(a)	1.43	1.31
Fe_3_O_4_-H@hpbtz(b)	1.11	2.54
Fe_3_O_4_-C(c)	1.02	1.21
Fe_3_O_4_-C@hpbtz(d)	6.07	5.41
Fe_3_O_4_@hpbtz(e)	2.61	8.73
MSN(f)	6.60	1.44 ^1^
MSN-NEt_3_-IPTMS-hpbtz-**f1**(g)	3.90	18.54 ^1^

^1^ 190–600 °C.

## Supplementary Materials

The following are available online at http://www.mdpi.com/2079-4991/8/6/434/s1: Figure S1., TGA thermograms of (a) Fe_3_O_4_-H, (b) Fe_3_O_4_-H@hpbtz, (c) Fe_3_O_4_-C, (d) Fe_3_O_4_-C@hpbtz, and (e) Fe_3_O_4_@hpbtz, using a heating rate of 20 °C min^−1^ in nitrogen; Figure S2., TGA thermograms of (f) MSNs and (g) MSN-Et_3_N-IPTMS-hpbtz-**f1**, using a heating rate of 20 °C min^−1^ in nitrogen; Figure S3., FTIR spectra of FMNPs (a) Fe_3_O_4_-H, (b) Fe_3_O_4_-C, (c) **hpbtz** ligand, (d) Fe_3_O_4_-H@hpbtz, (e) Fe_3_O_4_-C@hpbtz, and (f) Fe_3_O_4_@hpbtz; Figure S4., FTIR spectra of (a) MSNs and FMSNs (b) MSN-Et_3_N-IPTMS-hpbtz-**f1**, (c) MSN-Et_3_N-NCO-hpbtz–**e1**, (d) MSN-pyridine-IPTMS-hpbtz–**f2**, (e) MSN-pyridine-NCO-hpbtz-**e2**, (f) MSN-NaOH-IPTMS-hpbtz-**f3**, (g) MSN-NaOH-NCO-hpbtz-**e3,** and (h) **hpbtz** ligand; Figure S5., UV-vis spectra of MNPs, FMNPs, and **hpbtz** ligand at a concentration of 2 mg mL^−1^; Figure S6., UV-vis spectra of MSNs and FMSNs (a) MSNs, (b) MSN-Et_3_N-IPTMS-hpbtz-**f1**, (c) MSN-pyridine-IPTMS-hpbtz-**f2**, (d) MSN-NaOH-IPMS-hpbtz-**f3**, at a concentration of 2 mg mL^−1^, and (e) **hpbtz** ligand (10^−5^ M) [inset: c at a higher concentration]; Figure S7., ^1^H NMR spectrum of **hpbtz**; Figure S8., ^1^H NMR spectrum of **hpbtz**; Scheme S1., Synthetic method for the preparation of 2(2-hydroxyphenyl)benzothiazole (hpbtz) and the description of the benign synthesis of 2(2-hydroxyphenyl)benzothiazole (hpbtz).
