# Supplementary material for: Applications of Nanomaterials Based on Magnetite and Mesoporous Silica on the Selective Detection of Zinc Ion in Live Cell Imaging
Source: Nanomaterials (Basel). 2018 Jun 14;8(6):434. doi: 10.3390/nano8060434 (PMC6027406; doi:10.3390/nano8060434)
Supplement: Supplementary file 1 [file nanomaterials-08-00434-s001.pdf]

Applications of nanomaterials based on magnetite and mesoporous silica on the selective detection of zinc ion in live cell imaging

Roghayeh Sadeghi Erami[1,2], Karina Ovejero[3], Soraia Meghdadi[1], Marco Filice[3,4,5*], Mehdi Amirnasr[1,*], Antonio Rodríguez-Diéguez,[6] M. Ulagares De La Orden[7] and Santiago Gómez-Ruiz[2,*]

1 Department of Chemistry, Isfahan University of Technology, Isfahan 84156-83111, Iran; romochem@gmail.com (R.S.E.); smeghdad@cc.iut.ac.ir (S.M.)
2 Departamento de Biología y Geología, Física y Química Inorgánica, ESCET, Universidad Rey Juan Carlos, Calle Tulipán s/n, E-28933 Móstoles, Madrid, Spain
3 National Research Centre for Cardiovascular Disease (CNIC), Melchor Fernández Almagro, 3, 28029 Madrid, Spain; k.ovejero94@gmail.com
4 Dept. of Chemistry in Pharmaceutical Sciences, Faculty of Pharmacy, Complutense University (UCM), Plaza Ramón y Cajal s/n, 28040 Madrid, Spain
5 Biomedical Research Networking Center for Respiratory Diseases (CIBERES), Melchor Fernández Almagro, 3, 28029 Madrid, Spain
6 Departamento de Química Inorgánica, Facultad de Ciencias, Campus de Fuentenueva. Avda. Fuentenueva s/n, 18071 Granada, Spain; antonio5@ugr.es
7 Departamento de Química Orgánica I, E. U. Óptica, Universidad Complutense de Madrid, Arcos de Jalón, s/n, 28037 Madrid, Spain; mariula@quim.ucm.es
* Correspondence: mfilice@ucm.es (M.F.); amirnasr@cc.iut.ac.ir (M.A.); santiago.gomez@urjc.es (S.G.-R.); Tel.: +34-91-394-1751 (M.F.); +98-31-3391-2351 (M.A.); +34-91-488-8507 (S.G.-R.);
Fax: +98-31-3391-2350 (M.A.); +34-91-488-8143 (S.G.-R.)

Table of Contents – Supporting Information

1. Additional data of the characterizations of FMNPs and FMSNs 1
2. Description of the benign Synthesis of 2(2-hydroxyphenyl)benzothiazole (hpbtz) 6
References 8

1. Additional data of the characterizations of FMNPs and FMSNs

The thermal behavior of magnetite- and silica-based materials were investigated by thermogravimetric analysis (Figs. S1 and S2) and the resulting data are given in Table 3 of the manuscript. As shown in the thermograms of a-e (Fig. S1), the weight loss below 150 °C is characteristic of the adsorbed water and residual organic solvent in the magnetite-based materials. Moreover, MNPs and FMNPs exhibit an additional weight loss between around 190 to 450 °C. This step with 1.31, 2.54, 1.21, 5.41 and 8.73% weight loss for a to e, respectively, is attributed to the removal of organic moieties (including ethylene glycol, ascorbic acid and **hpbtz**) on the nanoparticles surface. As it is clear from the thermal analysis data, **Fe_3O_4-C@hpbtz** (d) has the highest weight loss compared to the other magnetite-based materials due to its higher organic content. However, the thermogram of e shows a weight loss between 190 to 450 °C including three steps presumably due to the decomposition of different organic fractions composed of ascorbic acid, as stabilizing agent, **hpbtz** ligand and residual ethylene glycol grafted on the nanoparticles surface. Fig. S2

presents the thermogravimetric analysis of MSNs and MSN-NEt3-IPTMS-hpbtz-**f1** (labeled as g and f, respectively). The initial weight loss up to 190 ºC in g and f is assigned to the desorption of residual water molecules trapped in the silica porous structure. The next main weight loss at above 200ºC is probably due to the decomposition of the organic fractions. It seems that the main weight loss with about 1.44% in the thermogram of MSNs is originated from the condensation of silanol groups of the material as it is mainly observed above 550 ºC. Moreover, the amount of the immobilized organic fractions onto MSN-NEt3-IPTMS-hpbtz-**f1** is estimated to be 18.54 %, corresponding to the presence of CTAB, IPTMS and **hpbtz** ligand.

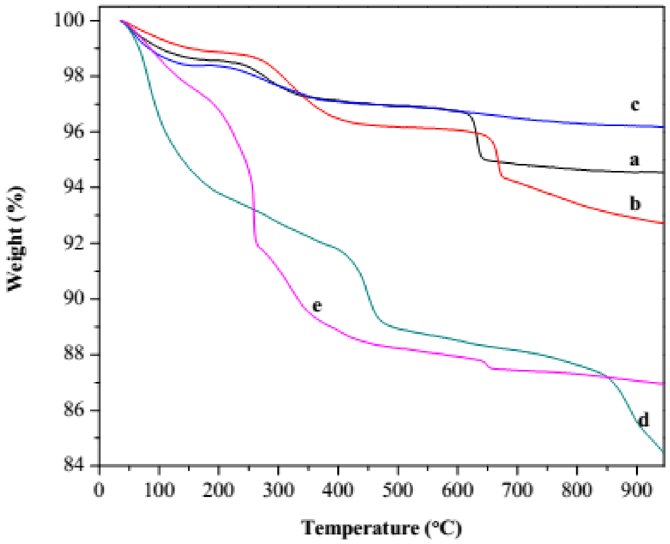

Figure S1. TGA thermograms of a) Fe_3O_4-H, b) Fe_3O_4-H@hpbtz, c) Fe_3O_4-C, d) Fe_3O_4-C@hpbtz and e) Fe_3O_4@hpbtz using a heating rate of 20 ºC min^{-1} in nitrogen.

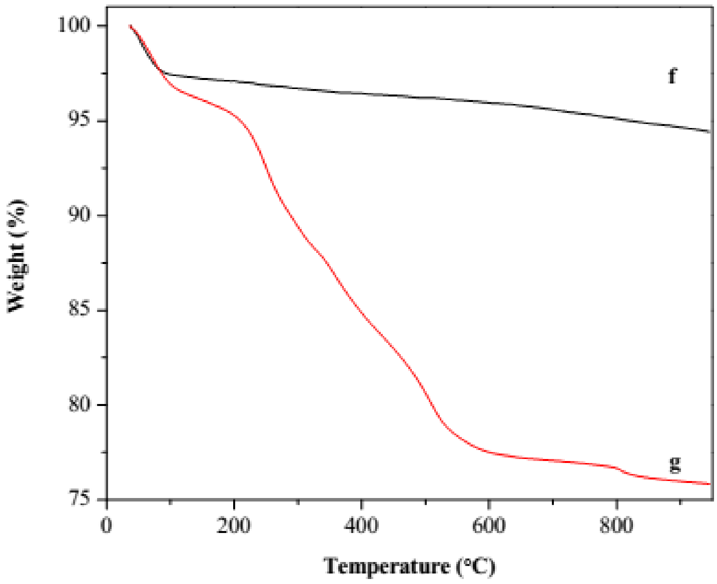

Figure S2. TGA thermograms of f) MSNs and g) MSN-Et_3N-IPTMS-hpbtz-**f1**, using a heating rate of 20 °C min^{-1} in nitrogen.

The introduction of the coupling agents and hpbtz fluorophore onto the magnetite and silica nanoparticles was further confirmed by FT-IR and UV-vis spectral analysis. The FT-IR spectra of FMNPs and FMSNs are presented in (Figs. S3 and S4). For the Fe3O4-H, Fe3O4-C, Fe3O4-H@hpbtz, Fe3O4-C@hpbtz, and Fe3O4@hpbtz (Fig. S3), there are two main bands at 570 cm−1 attributed to the vibration of Fe-O and a broad band at ca. 3430 cm-1 corresponds to the stretching of the O-H groups on the external NPs surface, indicating the magnetite formation. In addition, two bands at around 1630 and 1380 cm-1 are assigned to the bending vibration and deformation of the H-O-H of physically adsorbed water, respectively. In the spectrum of hpbtz (Fig. S3c), there are two characteristic bands at 1600 and 1580 cm-1 assigned to the (C=C) and (C=N) stretching vibrations and around 1487 and 1439cm-1 characteristic of the (C=C) stretching and (C-H) bending vibrations of the benzothiazole moiety [1]. The first two characteristic bands of hpbtz, i.e. azomethine (C=N) and (C=C) stretching vibrations appear in the nearly similar positions in the functionalized materials spectra (Fig S3 d, e, and f). In the S1f spectrum, the C=O stretching bands of vitamin C and its oxidized form are absent indicating that the carbonyl group is modified by coordinating to the Fe center on the MNPs surface [2,3]. The presence of low intensity peaks around 1400 cm-1 are also assigned to the aromatic C-H bending vibrations of the benzothiazole moiety in S3 d, e and f [4].

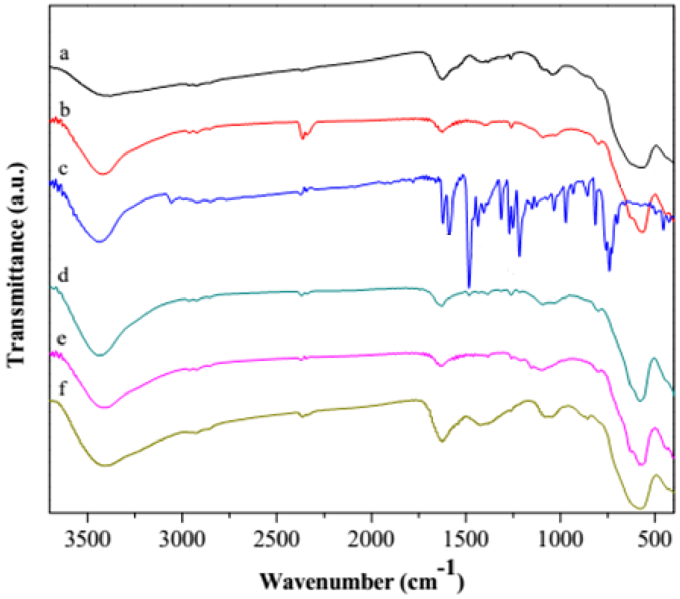

Figure S3. FT-IR spectra of FMNPs a) Fe_3O_4-H, b) Fe_3O_4-C, c) hpbtz ligand, d) Fe_3O_4-H@hpbtz, e) Fe_3O_4-C@hpbtz and f) Fe_3O_4@hpbtz.

The FT-IR spectra of MSNs and FMSNs: MSN-Et_3N-IPTMS-hpbtz-f1, MSN-pyridine-IPTMS-hpbtz-f2, MSN-NaOH-IPTMS-hpbtz-f3, MSN-Et_3N-NCO-hpbtz-e1, MSN-pyridine-NCO-hpbtz-e2, MSN-NaOH-NCO-hpbtz-e3 and hpbtz ligand are shown in **Fig. S4**. The FT-IR spectrum of MSNs (**Fig. S4, a**) shows characteristic band at 1630 cm^{-1} assigned to O-H stretching vibration of the remaining physisorbed water molecules. As a result of mesoporous silica formation, a strong peak with maximum near 1090 cm^{-1}, corresponding to Si-O-Si stretching vibrations appears. Two additional bands appearing at 970 and 804 cm^{-1} are due to Si-O-H and Si-O-Si bending vibrations. The IPTMS and ICTES functionalized materials (**Fig. S4, b-g**) display additional absorption bands at around 2930 and 2980 cm^{-1} due to the ν(C–H) stretching

vibrations in the propyl chains of organosilane coupling agents. In the **S4 c, e and g** spectra the bending vibration of amidic N-H appears at 1580 cm $^{-1}$. Moreover, the disappearance of the band at 2225 cm $^{-1}$ is attributed to the stretching vibration of NCO, and the appearance of a new carboxylic band at 1680 cm^{-1} indicate that the reaction between the isocyanate group in ICTES and hydroxyl of hpbtz occurred successfully [5,6]. However, the strong Si-O-Si stretching vibrations near to 1090 cm^{-1} attenuates the intensity of the characteristic bands of hpbtz which are expected to appear at around 1400 cm^{-1}.

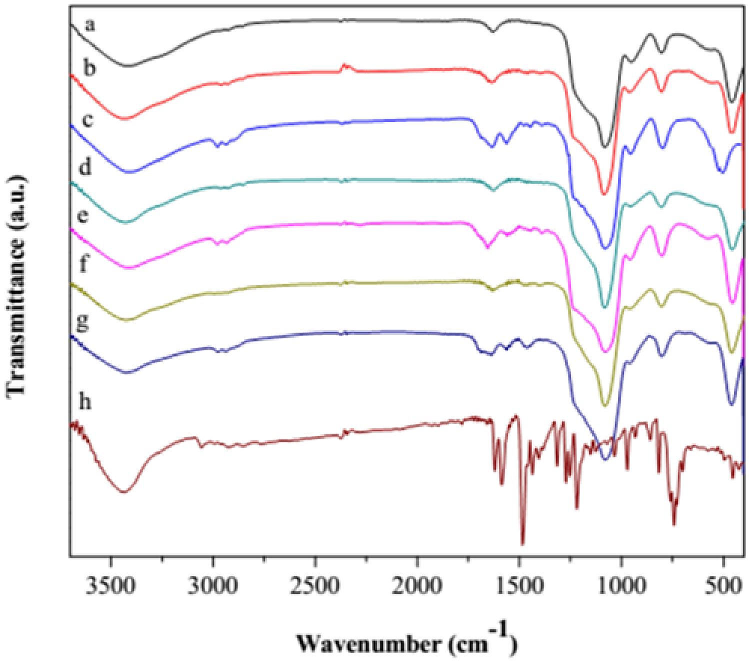

Figure S4. FT-IR spectra of a) MSNs and FMSNs: b) MSN-Et$_3$N-IPTMS-hpbtz-**f1**, c) MSN-Et$_3$N-NCO- hpbtz –**e1**, d) MSN-pyridine-IPTMS-hpbtz–**f2**, e) MSN-pyridine-NCO-hpbtz-**e2**, f) MSN-NaOH-IPTMS-hpbtz-**f3**, g) MSN-NaOH-NCO-hpbtz-**e3** and h)hpbtz ligand.

The UV-vis spectra of FMNPs and FMSNs are presented in (**Figs. S5 and S6**). **Fig. S5** shows a comparative UV-vis study of colloidal dispersion of MNPs and their functionlization with hpbtz at a concentration of 2 mg mL^{-1} in ethanol. The spectrum of pure hpbtz dissolved in ethanol is also given for comparison. The magnetite nanoparticles (Fe_3O_4-H and Fe_3O_4-C) spectra show a broad absorption band at around 350 nm, corresponding to the charge transfer from oxide to iron Fe^{3+} d orbitals in the octahedral and tetrahedral sites [7]. The absorption spectrum of hpbtz shows three absorption bands at 216, 288 and 336 nm corresponds to the intra ligand charge transitions [8].

From the comparison between the absorption spectra of hpbtz and FMNPs in **Fig. S5** it is clear that the incorporation of hpbtz onto the magnetite nanoparticles leads to a slight red shift in the ligand absorption bands. This is a good evidence for effective coordination of the ligand to the MNPs. Furthermore, a new broad band appears at around 400 nm in the Fe_3O_4-H@hpbtz, Fe_3O_4-C@hpbtz and Fe_3O_4@hpbtz spectra due to the ligand to metal charge transfer from the surface bound ligand to the metal core.

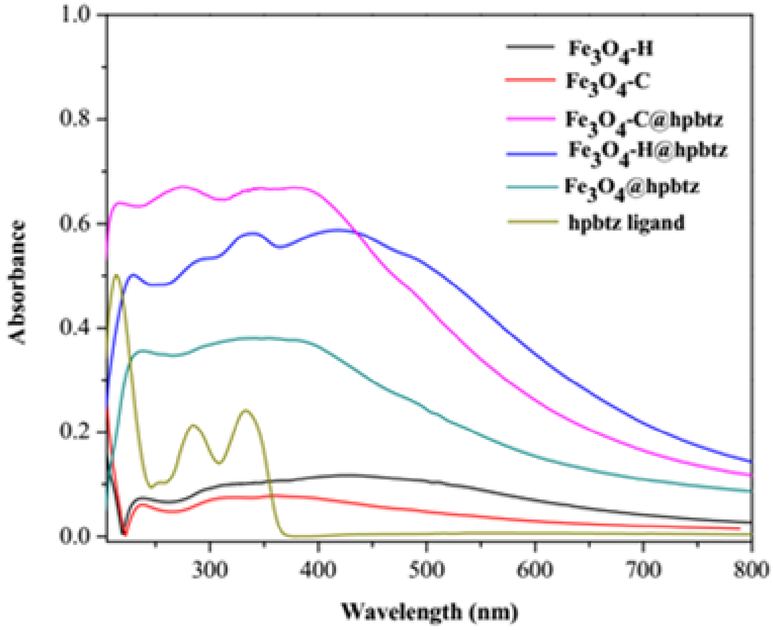

Figure S5. UV-vis spectra of MNPs, FMNPs and hpbtz ligand at a concentration of 2 mg mL-1.

Considering the higher ligand quantity in mmol per gram of material prepared in post functionalization with IPTMS, and determined by XRF analysis data (**Table 2**), comparative UV-vis spectra of colloidal dispersion of MSN and FMSNs (f1-f3) at a concentration of 2 mg mL^{-1} in ethanol have been recorded (**Fig. S6**). The spectrum of pure hpbtz dissolved in ethanol is also given for comparison (**Fig S6, e**). For mesoporous silica nanoparticles there are two characteristic bands at 223 and 288 nm correspond to Si-O ligand to metal charge transfer. As shown in **Figs. S6, b, c** and **d**, the spectra of functionalized mesoporous materials show characteristic absorption bands from hpbtz and correspond to the intraligand transitions.

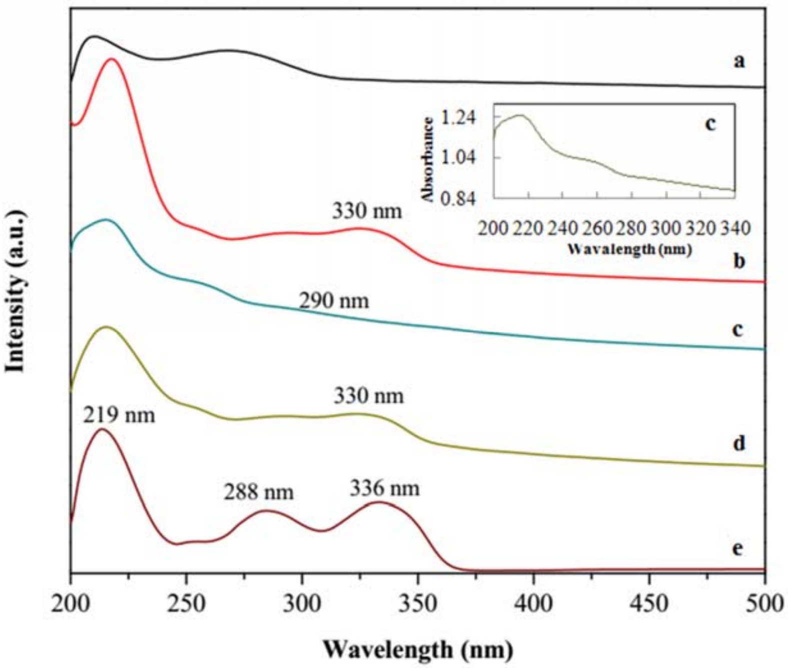

Figure S6. UV-vis spectra of MSNs and , FMSNs: a)MSNs, b) MSN-Et_3N-IPTMS-hpbtz-**f1**, c) MSN-pyridine-IPTMS-hpbtz-**f2,**d) MSN-NaOH-IPMS-hpbtz-**f3**, at a concentration of 2 mg $mL^{-1}$and e) hpbtz ligand (10^{-5} M).[inset: c at a higher concentration].

2. Description of the benign Synthesis of 2(2-hydroxyphenyl)benzothiazole (hpbtz)

The ligand hpbtz was synthesized by a benign and environmentally friendly method that we have recently reported using the ionic liquid tetrabutylammonium bromide (TBAB) as the reaction medium [9]. Briefly, as show in **Scheme S1**, a mixture of triphenyl phosphite (TPP) (1.55 g, 5 mmol), tetrabutylammonium bromide (TBAB) (1.66 g, 5 mmol), 2-aminothiophenol (0.625 g, 5 mmol) and 2-hydroxybenzoic acid (0.69 g, 5 mmol) in a 25 mL round bottomed flask was placed in an oil bath. The solution was stirred for 60 min at 120°C. The product precipitated from the viscous solution by adding MeOH, and the resulting solid was filtered off and washed with cold MeOH.Yield:76 mg, 92%, ^{1}H NMR: Figs**. S7-S8.**

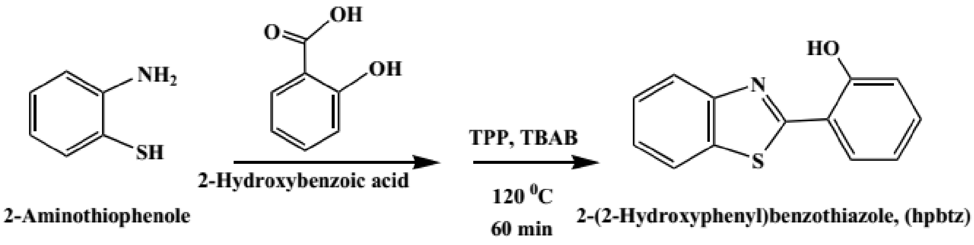

Scheme S1. Synthetic method for the preparation of 2(2-hydroxyphenyl)benzothiazole (hpbtz).

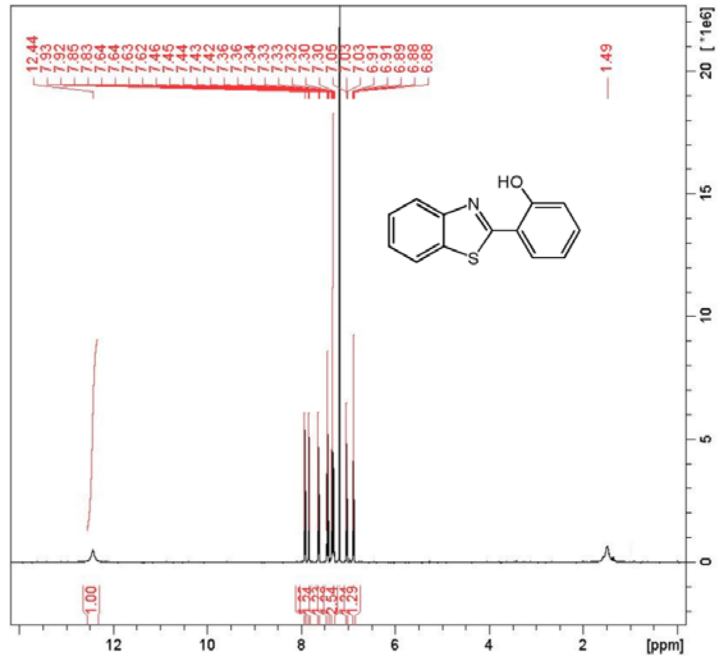

Figure S7. 1H NMR spectrum of Hpbtz

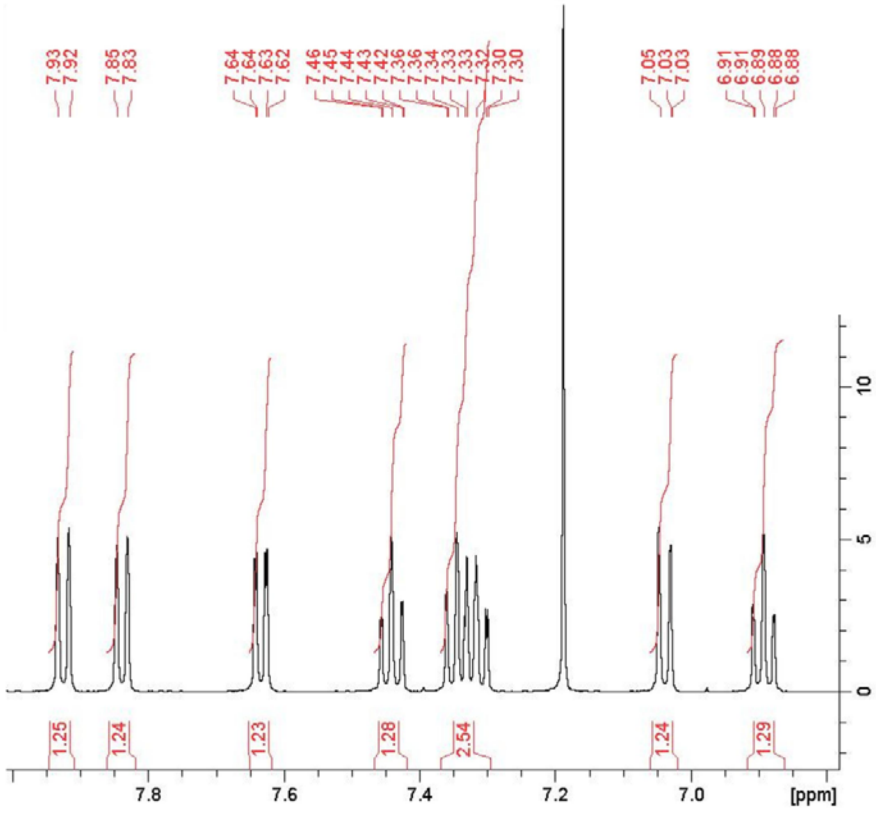

Figure S8. ^{1}H NMR spectrum of Hpbtz

References

1. Li, Z.; Dellali, A.; Malik, J.; Motevalli, M.; Nix, R.M.; Olukoya, T.; Peng, Y.; Ye, H.; Gillin, W.P.; Hernandez, I.; Wyatt, P.B. Luminescent Zinc(II) Complexes of Fluorinated Benzothiazol-2-yl Substituted Phenoxide and Enolate Ligands. *Inorg. Chem*. **2013**, 52, 1379–1387, DOI: 10.1021/ic302063u.
2. Li, Z.; Chen, H.; Bao, H., Gao, M. One-Pot Reaction to Synthesize Water-Soluble Magnetite Nanocrystals. *Chem. Mater.***2004**, 16, 1391–1393, DOI: 10.1021/cm035346y.
3. Li, Z.; Wei, L.; Gao, M.; Lei, H. One-Pot Reaction to Synthesize Biocompatible Magnetite Nanoparticles. *Adv. Mater*. **2005**, 17, 1001–1005, DOI: 10.1002/adma.200401545.
4. Nakamoto, K. *Infrared and Raman spectrum of Inorganic and Coordination Compounds*, fifth ed., John Wiley and Sons Inc., New York, 1997, DOI: 10.1002/9780470405840.
5. Saleh, T.A. The influence of treatment temperature on the acidity of MWCNT oxidized by HNO3 or a mixture of HNO_3/H_2SO_4. *Appl. Surf. Sci*, **2011**, 257, 7746-7751, DOI: 10.1016/j.apsusc.2011.04.020.
6. Gupta, V.K.; Agarwal, S.; and Saleh, T.A. *J. Hazard. Mater*. **2011**, 185, 17-23, DOI: 10.1016/j.jhazmat.2010.08.053.
7. Sherman, D.M.; Waite, T.D. Electronic spectra of Fe^{3+} oxides and oxide hydroxides in the near IR to near UV. *Am. Mineral*. **1985**, 70, 1262-1269.
8. Grando, S.R.; Pessoa, C.M.; Gallas,M.R.; Costa,T.M.H.; Rodembusch, F.S.; and Benvenutti, E.V. Modulation of the ESIPT Emission of Benzothiazole Type Dye Incorporated in Silica-Based Hybrid Materials. *Langmuir* **2009**, 25, 13219–13223, DOI: 10.1021/la902242y.
9. Meghdadi, S.; Amirnasr, M.; and Ford, P.C. A robust one-pot synthesis of benzothiazoles from carboxylic acids including examples with hydroxyl and amino substituents. *Tetrahedron Lett*. **2012**, 53, 6950–6953, DOI: 10.1016/j.tetlet.2012.10.035.
